# Natural Product‐Induced Ferroptosis to Overcome Therapy Resistance in Aggressive Cancers

**DOI:** 10.1002/cmdc.70548

**Published:** 2026-10-26

**Authors:** Giorgio Antoniolli

**Affiliations:** ^1^ Institute of Chemistry University of São Paulo São Paulo Brazil; ^2^ Institute of Environmental, Chemical and Pharmaceutical Sciences Federal University of São Paulo Diadema Brazil

**Keywords:** cancer therapy, ferroptosis, GPX4 inhibition, natural products, polypharmacology

## Abstract

Ferroptosis as an emerging control for cell death: ferroptosis was introduced as an advanced version of regulated cell death that is defined by iron‐dependent lipid peroxidation and redox imbalance, providing a candidate for anticancer medicine because of the possibility to achieve reduction of cancer resistance with conventional therapeutic agents. Recent findings demonstrate that natural products are a prospective rich source of ferroptosis regulators through their structural variety and intrinsic redox activity. In this perspective, we present a brief overview of recent developments (from 2026 to 2021) in natural product‐induced ferroptosis and their translation into cancer treatments. Ferroptosis has been initiated via glutathione depletion, glutathione peroxidase 4 inhibition, iron accumulation, and generation of reactive oxygen species by natural compounds, such as polyphenols, alkaloids, terpenoids, and quinones. Several natural products show remarkable susceptibility to ferroptotic vulnerabilities that can sensitize drug‐resistant cancer cells to chemotherapy and radiotherapy. Moreover, the polypharmacological characteristic of natural scaffolds allows the simultaneous manipulation of interrelated metabolic and redox pathways, which enhances therapeutic efficacy. Nevertheless, bioavailability, selectivity, and mechanistic comprehension are still challenges for such interesting discoveries. Natural product‐induced ferroptosis in general is an attractive and novel alternative for promoting novel anticancer therapeutic strategies.

## Graphical abstract

Natural products trigger ferroptosis through iron overload, glutathione depletion, glutathione peroxidase 4 inhibition, and reactive oxygen species accumulation, selectively targeting aggressive, and drug‐resistant cancers. Polyphenols, alkaloids and terpenoids emerge as promising multi‐target agents to overcome therapy resistance and improve future anticancer strategies.

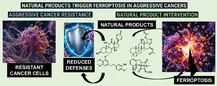

**Abbreviations**
4‐HNE: 4‐hydroxynonenal
ACSL4: acyl‐CoA synthetase long‐chain family member 4
AI: artificial intelligence
AKR1C1: aldo‐keto reductase family 1 member C1
AML: acute myeloid leukemia
AMPK: AMP‐activated protein kinase
APEX1: apurinic/apyrimidinic endodeoxyribonuclease 1
AR: androgen receptor
ATG7: autophagy related 7
ATP1A1: ATPase Na^+^/K^+^ transporting subunit Alpha 1
BCL‐2: B‐cell lymphoma 2
BSO: buthionine sulfoximine
CA: centrosome amplification
CCL2: C–C motif chemokine ligand 2
CETSA: cellular thermal shift assay
CM: conditioned medium
CoQ10: coenzyme Q10
CRISPR: clustered regularly interspaced short palindromic repeats
CRPC: castration‐resistant prostate cancer
DARTS: drug affinity responsive target stability
DFO: deferoxamine
DHODH: dihydroorotate dehydrogenase
EGFR‐TKI: epidermal growth factor receptor‐tyrosine kinase inhibitor
EMT: epithelial‐mesenchymal transition
ER: endoplasmic reticulum
ETS1: E26 transformation specificity‐1
FIN56: *N* ^2^,*N* ^7^‐Dicyclohexyl‐9‐(hydroxyimino)‐9*H*‐fluorene‐2,7‐disulfonamide
FOSL2/FOXC1: FOS like 2, AP‐1 transcription factor subunit/forkhead box C1
FSP1: ferroptosis suppressor protein 1
FTH1: ferritin heavy chain 1
FUNDC1: FUN14 domain‐containing protein 1
GCH1: GTP cyclohydrolase 1
GCS: γ‐glutamylcysteine synthetase
GES‐1: gastric epithelial cells
GPX4: glutathione peroxidase 4
GSH: glutathione
GSTM3: glutathione S‐transferase mu 3
HCC: hepatocellular carcinoma
HDI: human development index
Hic1: hypermethylated in cancer 1
HMOX1: heme oxygenase 1 gene
HO‐1: heme oxygenase‐1
HSP70: heat shock protein 70
IAP: inhibitor of apoptosis proteins
IC_50_
: half maximal inhibitory concentration
iNOS: inducible nitric oxide synthase
JNK: C‐Jun N‐terminal kinase
KIF11: kinesin family member 11
KRAS: kirsten rat sarcoma viral oncogene homolog
LC3B: microtubule‐associated protein 1 light chain 3 Beta
LOXs: lipoxygenases
MDA: malondialdehyde
MEF: mouse embryonic fibroblast
NCOA4: nuclear receptor coactivator 4
Nedd4: neural precursor cell expressed developmentally downregulated 4
NKTCL: natural killer/T‐cell lymphoma
NQO1: NAD(P)H quinone dehydrogenase 1
NRF2: nuclear factor erythroid 2‐related factor 2
NSCLC: nonsmall cell lung cancer
PARP1: poly(ADP‐ribose) polymerase 1
PBS: phosphate‐buffered saline
PCBP1/2: poly(rC)‐binding proteins 1/2
PD‐1: programmed cell death protein 1
PD‐L1: programmed cell death ligand 1
PERK: protein kinase R‐like endoplasmic reticulum kinase
PINK1: PTEN‐induced kinase 1
PKCδ: protein kinase C δ
PLA2: phospholipase A2
PLOOHs: lipid hydroperoxides
PRDX1: peroxiredoxin 1
PRDX2: peroxiredoxin 2
PRDX5: peroxiredoxin‐5
PUFA: polyunsaturated fatty acid
RCD: regulated cell death
RORC: related orphan receptor C
ROS: reactive oxygen species
RSL3: (1*S,*3*R*)‐*N*‐(3‐methoxyphenyl)‐3‐methyl‐2,3‐dihydro‐1,4‐benzoxazine‐6‐sulfonamide
RUNX2: runt‐related transcription factor 2
SCLC: small cell lung carcinoma
SLC3A2: solute carrier family 3 member 2
SLC7A11: solute carrier family 7 member 11
SOD1: superoxide dismutase 1
STAT3: signal transducer and activator of transcription 3
SYVN1: synoviolin 1
TCA: tricarboxylic acid cycle
TFR1: transferrin receptor 1
TFRC: transferrin receptor
TNBC: triple‐negative breast cancer
TRIM26: tripartite motif containing 26
TXNRD1: thioredoxin reductase 1
VDAC2: voltage‐dependent anion channel 2
WWVOX: WW domain‐containing oxidoreductase

## 1 Introduction

Cancer is still one of the top causes of morbidity and mortality globally, with aggressive and drug‐resistant tumors being a pressing clinical issue [1, 2]. Despite massive advances in targeted therapies and immunotherapy, the emergence of resistance to traditional therapies has hampered long‐term therapeutic efficacy and consequently, is responsible for poor patient prognosis. Especially, many tumors gain resistance to chemotherapy due to mechanisms such as increased antioxidant defenses, metabolic reprogramming, and evasion of programmed cell death pathways [2, 3]. In general, the emergence of both intrinsic and acquired chemoresistance via changes in genetic and epigenetic factors contributes significantly to treatment failure across multiple cancer types [4, 5, 6]. The development of combination therapy is a sensible way to reduce drug‐resistant cancer cells. By combining drugs with different modes of action and nonoverlapping resistance pathways, this strategy minimizes selective pressure and increases the efficacy of such agents (Figure 1). These challenges highlight the pressing requirement to develop new drugs that can address these limitations and, at the same time, afford more efficacy and greater tolerability for patients.

**FIGURE 1:**
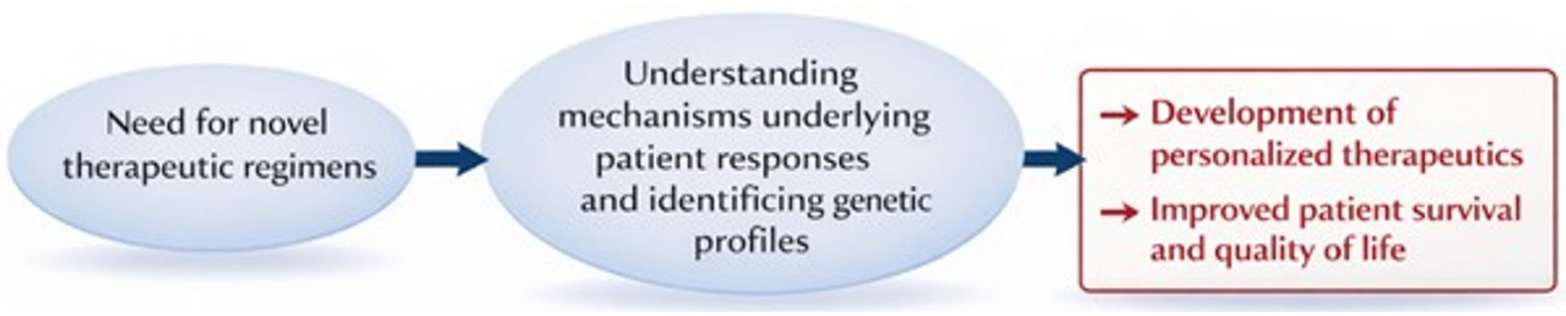
A personalized therapeutic framework highlights the need for new anticancer agents. As resistance, toxicity, and reduced efficacy limit current treatments, advancing research at the interface of medicinal chemistry and oncology remains essential for developing safer and more effective therapies.

In 2022, global cancer statistics show approximately 20 million newly identified cases (including non‐melanoma skin cancers) and around 9.7 million cancer deaths globally. Lung cancer ranks as the most common cancer, and it accounted for nearly 2.5 million new cases (12.4%) of cancers in the world, followed by female breast (11.6%) and colorectal (9.6%) cancers, prostate (7.3%) cancers, and stomach cancers (4.9%). In terms of mortality, lung cancer was also the top factor responsible for cancer death, with an estimated 18.7% of deaths (∼1.8 million), followed by 9.3% for colorectal, 7.8% for liver, 6.9% for female breast, and 6.8% for stomach cancers. Melanoma had a relatively lower rate of disease at around 3.7% in males and 2.9% in females. Demography‐based estimates indicate that the worldwide burden of cancer is expected to increase significantly, reaching around 35 million new cases by 2050, an increase of about 77% as opposed to 2022 [1, 2].

In Brazil, it is projected that there will be around 704,000 new cancer cases in 2023, including nonmelanoma skin cancers. If we omit those, we’re referring to some 483,000 new cases. Among these types of cancer, female breast cancer and prostate cancer account for about 15% of all cases, whereas colorectal cancer accounts for 9.4%, trachea, bronchus, and lung cancers for 6.7%, stomach cancer accounts for 4.4%, and cervical cancer accounts for 3.5%. Now we break this down into gender: prostate cancer is the most common cancer among men (30%), followed by colorectal cancer (9.2%). Breast cancer is the most common cancer in women, accounting for 30.1% of diagnoses, and colorectal cancer (9.7%) is a relatively close second. Cancer disease and disease incidence are similar in Brazil to countries based on a high human development index (HDI), with breast, prostate, colorectal, lung, cervical, stomach, and oral cavity cancers leading the statistics. However, the way things play out is complicated. In addition to these cancers frequently associated with westernized lifestyles, Brazil is also facing a considerable burden of cancers linked to infections and poverty, including cervical and stomach cancers. This blend has mirrored the continuing socioeconomic and demographic transitions in the nation [7, 8, 9].

Traditional anticancer treatments, such as most chemotherapeutics and radiotherapy, are mainly aimed at killing malignant cells by activating apoptotic pathways. However, the escape from apoptosis as a cancer signature and principal cause of treatment‐related failure and relapse is now well documented. Tumor cells frequently acquire intricate changes in the apoptotic machinery in general, such as TP53 loss or alteration, repression of anti‐apoptotic B‐cell lymphoma 2 (BCL‐2) family as well as inhibitor of apoptosis (IAP) proteins, death receptor inhibition or downregulation, and aberrant initiation or effector caspase activation, which increase the threshold to apoptosis and confer multidrug resistance. Such adaptations may not only blunt the cytotoxic effect of apoptosis‐inducing agents but also induce immune escape and tumor progression [10, 11, 12, 13]. As a result, the therapeutic application of alternative regulated cell death (RCD) modalities, such as necroptosis, ferroptosis, pyroptosis, parthanatos, and cuproptosis, to kill apoptosis‐refractory cancer cells and in some cases even modulate the tumor microenvironment toward enhancing antitumor immunity and overcoming drug resistance, has attracted increasing interest [14, 15, 16, 17].

Ferroptosis has thus emerged as a mechanistically and morphologically different regulated type of cell death characterized by iron‐dependence in the accumulation and oxidation of phospholipid peroxides with subsequent destruction of cellular membranes [18, 19, 20, 21, 22]. In contrast to caspase‐dependent apoptosis, ferroptosis results from metabolic and redox pathology comprising cysteine/glutathione deprivation, loss of activity of the lipid repair enzyme glutathione peroxidase 4 (GPX4), and an accumulation of lipid‐derived reactive oxygen species (ROS) produced by iron‐mediated Fenton chemistry and polyunsaturated phospholipid peroxidation [20, 21, 23, 24, 25, 26, 27]. This death program is even more finely tuned by concurrent antioxidant systems with complementary mechanisms, such as the system X_c_^−^–GSH (glutathione)–GPX4 axis, ferroptosis suppressor protein 1–Coenzyme Q10 (FSP1–CoQ10), and dihydroorotate dehydrogenase (DHODH)–mediated mitochondrial defense, which buffer cytotoxic lipid peroxides [18, 23, 25, 28, 29, 30, 31].

Cancer cells often exhibit reprogrammed iron metabolism, with high polyunsaturated lipid contents, and reorganized antioxidant circuits, leaving mesenchymal, dedifferentiated, and therapy‐resistant clones particularly vulnerable to ferroptotic stress [18, 32, 33, 34, 35]. Therefore, pharmacological induction of ferroptosis, either through GPX4 or system X_c_^−^ inhibition, disruption of CoQ10‐based defense, or targeting metabolic nodes capable of augmenting lipid peroxidation, has become a powerful approach to exploit tumor‐specific metabolic and redox vulnerabilities and to overcome resistance to chemotherapy, radiotherapy, and targeted agents (Figure 2) [18, 19, 22, 28, 32, 36].

**FIGURE 2:**
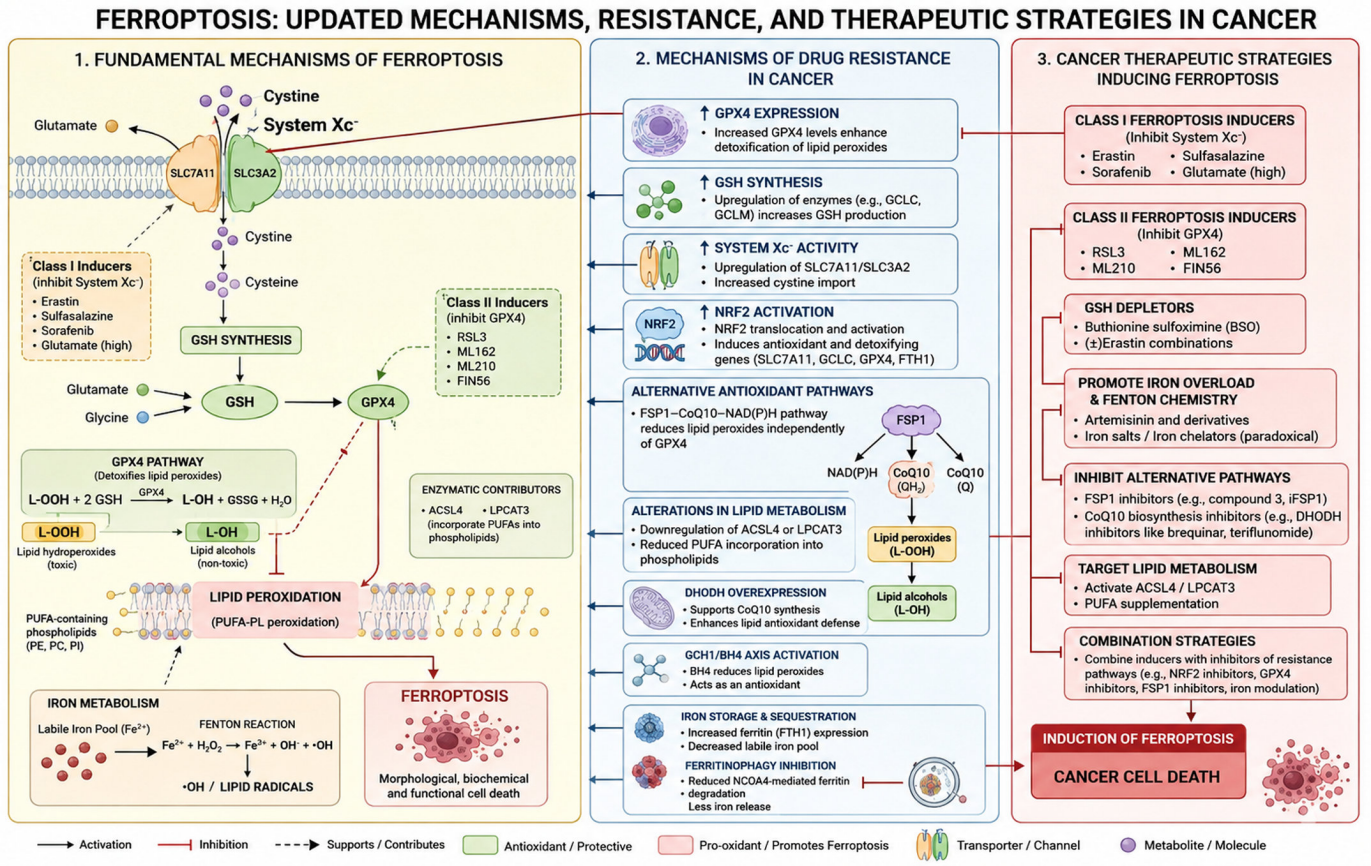
A detailed schematic representation of ferroptosis, an iron‐dependent, nonapoptotic form of cell death in the context of cancer, highlighting its core mechanisms, resistance pathways, and therapeutic strategies. The diagram is organized into three interconnected panels depicting the canonical molecular pathway, adaptive responses in cancer cells, and points of therapeutic intervention. This image was generated using artificial intelligence (Gemini 3, reasoning function) and subsequently fully edited.

Small‐molecule drugs targeting ferroptosis have been promising anticancer strategies for targeting critical regulatory pathways. Sorafenib and sulfasalazine, for example, induce ferroptosis by inhibiting system X_c_^−^, and altreta‐mine and (1*S*,3*R*)‐*N*‐(3‐methoxyphenyl)‐3‐methyl‐2,3‐dihydro‐1,4‐benzoxazine‐6‐sulfonamide (RSL3) increase lipid peroxidation by suppressing GPX4 activity. Statins indirectly impair GPX4 biosynthesis through blockade of the mevalonate pathway, and *N* ^2^,*N* ^7^‐Dicyclohexyl‐9‐(hydroxyimino)‐9*H*‐fluorene‐2,7‐disulfonamide (FIN56) promotes ferroptotic cell death by driving degradation of GPX4 and depleting coenzyme Q10. Also, tyrosine kinase inhibitors like lapatinib and neratinib lead to increased intracellular iron availability and thus ferroptosis. Erastin, for example, works via various mechanisms, such as inhibition of solute carrier family 7 member 11 (SLC7A11) and modulation of mitochondrial outer membrane permeability. Although some of these agents have clinical approval for other indications, their utility as ferroptosis‐targeted therapeutics in oncology remains under active investigation (Figure 3) [37, 38].

**FIGURE 3:**
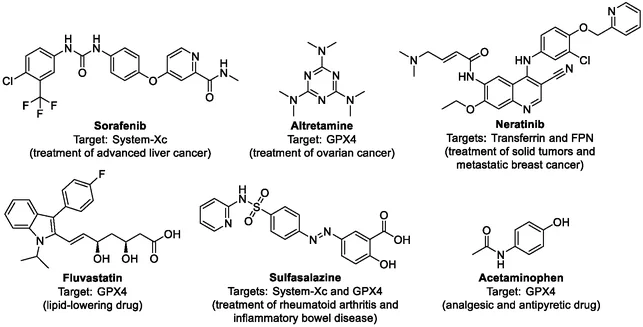
Approved small‐molecule drugs targeting ferroptosis in cancer therapy.

Natural products have been the cornerstone of anticancer drug discovery for decades, providing a variety of structurally diverse scaffolds with broad bioactivity and multi‐target engagement [39, 41] (Figure 4). Their inherent complexity makes them particularly ideal as an alternative target for modulating complex networks regulating redox, iron metabolism, and RCD (including ferroptosis) [32, 42, 43]. Recent evidence has also shown that many plant‐ and marine‐derived compounds, including terpenoids, polyphenols, alkaloids, and quinones, can induce or inhibit ferroptosis by acting upon important nodes of the ferroptotic machinery [40, 44, 45]. From a mechanistic perspective, these molecules mainly act by altering iron homeostasis and ferritinophagy, blocking the cyst(e)ine–GSH–GPX4 antioxidant axis, modulating nuclear factor erythroid 2‐related factor 2/heme oxygenase‐1 (NRF2/HO‐1) signaling, or lethally inducing the peroxidation of polyunsaturated phospholipids [32, 45, 46, 47]. Taking advantage of tumor‐specific iron and redox buffering, ferroptosis‐modulating natural products are gaining increasing interest as means of circumventing therapy resistance as well as changing the tumor microenvironment in a variety of cancers [32, 40, 43, 47].

**FIGURE 4:**
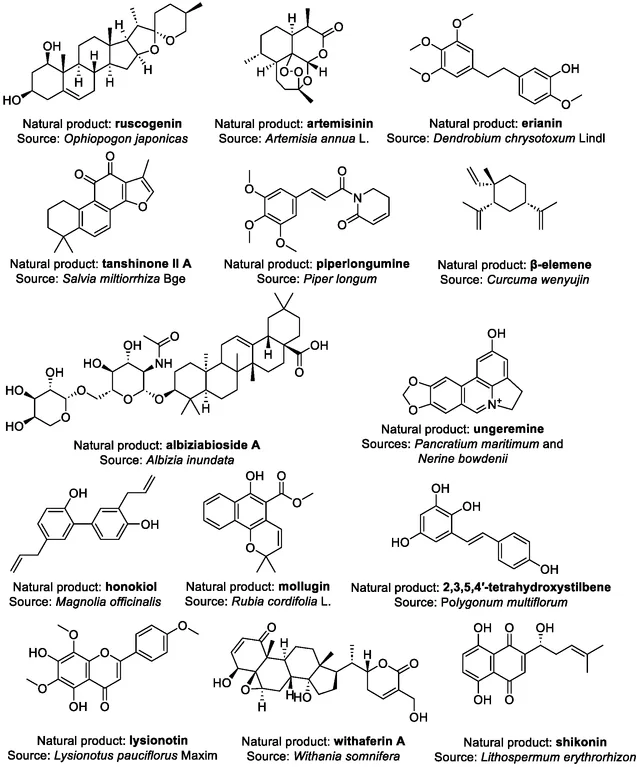
Natural compounds modulating ferroptosis pathways.

In the last decade, the research topic of ferroptosis has developed rapidly, and natural products have become a particularly rich line of small‐molecule agents to induce or inhibit the death of ferroptotic cells [43, 45, 48]. Chemoinformatic and systematic reviews have reported several hundred modulators of ferroptosis, many natural by‐products or semisynthetic derivatives, out of which at least several dozen to >60 individual natural compounds have been reported to modulate ferroptosis in tumor models [37, 39, 43]. These agents range in structure from polyphenols, terpenoids, alkaloids, quinones, and marine metabolites and use many, often concordant mechanisms, such as iron handling and ferritinophagy regulation, interference at the System X_c_^−^–GSH–GPX4 axis, and upregulation or downregulation of lipid peroxidation and redox signaling [44, 46]. The rapidly advancing catalog of these natural ferroptosis inducers and inhibitors highlights their worth as privileged scaffold material for anticancer drug discovery and especially for overcoming therapy resistance, enabling rational ferroptosis‐based combination strategies [32, 40, 47].

Therefore, targeting ferroptotic vulnerabilities with natural products represents a compelling and innovative strategy to overcome therapy resistance in aggressive cancers. By exploiting tumor‐specific metabolic dependencies, iron addiction, and redox disequilibria, natural compounds may open a new avenue for the development of effective anticancer therapies that circumvent the limitations of conventional apoptosis‐centered approaches. In this context, ferroptosis‐inducing natural products are particularly attractive as they can selectively eliminate malignant cells that have acquired resistance to genotoxic agents while sparing normal tissues. This review focuses on natural compounds capable of triggering or sensitizing ferroptosis and discusses their potential to overcome therapeutic resistance and to be integrated into rational combination regimens in difficult‐to‐treat malignancies.

## 2 Molecular Basis of Ferroptosis

Ferroptosis is a distinct form of RCD driven by iron‐dependent lipid peroxidation and the accumulation of lethal ROS. Ferroptosis is an alternative pathophysiological process to apoptosis, necrosis, or autophagy and can be distinguished mainly through the modulation of metabolic and redox homeostasis via processes in iron metabolism, lipid remodeling, and antioxidant defense. These intertwined events together influence the cellular vulnerability to ferroptotic damage and are important targets of therapeutic interventions. Iron is central for ferroptosis as it is an efficient catalyst for the production of highly reactive radicals via Fenton chemistry. In cancer cells, the iron metabolism is frequently dysregulated, leading to increased activity of transferrin receptor 1 (TFR1), reduced ferritin storage, and increased labile iron pools. The iron overload stimulates the production of hydroxyl radicals (•OH) that lead to lipid peroxidation cascades and oxidative membrane damage. Moreover, autophagic degradation of ferritin (ferritinophagy) expands the cellular availability of iron from the inside and sensitizes cells to ferroptosis. These changes are especially apparent in aggressive tumors, for which iron metabolism is closely associated with growth and metabolic rearrangement [49, 50].

Ferroptosis is when the polyunsaturated fatty acid (PUFA)‐containing phospholipids within cellular membranes undergo oxidation and peroxidation. This mechanism is initiated by ROS and enzymatically regulated by lipid metabolism enzymes, such as acyl‐CoA synthetase long‐chain family member 4 (ACSL4), which facilitates the incorporation of PUFAs into phospholipids. Once oxidized by these lipids, either via nonenzymatic radical reactions or lipoxygenases (LOXs), lipid hydroperoxides (PLOOHs) were formed. Upon reaching an intensity of peroxidation beyond the capacity of cellular antioxidant systems to detoxify peroxidized lipids, they induce membrane instability and loss of integrity and thus generate ferroptotic cell death [18, 51].

The main cell defense against ferroptosis consists of the cystine/glutamate antiporter system X_c_^−^ (composed of SLC7A11/solute carrier family 3 member 2 (SLC3A2)) and glutathione (GSH)‐dependent enzyme GPX4. System X_c_^−^ takes in cystine, which is subsequently reduced to cysteine to make GSH. GSH is a crucial cofactor for GPX4, which allows the reduction of lipid hydroperoxides to nontoxic lipid alcohols. Both the inhibition of system X_c_^−^ and the depletion of intracellular GSH compromise GPX4 activity, and induce lipid peroxide accumulation and ferroptosis. Because of its central role, GPX4 is considered a master regulator of ferroptotic cell death and an important target for therapeutics. Notably, a lot of ferroptosis‐inducing agents, such as natural products, work on this axis by interrupting it [18, 27].

Even though ferroptosis is not strictly dependent upon mitochondria, the role of mitochondrial metabolism in ferroptotic sensitivity is being recognized as a potential factor, as demonstrated by a growing body of evidence. Mitochondria are significant producers of ROS, particularly under conditions of metabolic stress, and can amplify oxidative damage through electron transport chain dysfunction. Moreover, changes in mitochondrial membrane potential, tricarboxylic acid cycle (TCA) activity, and oxidative phosphorylation may stimulate lipid peroxidation and ferroptotic signaling. Combining mitochondrial ROS production and cytosolic antioxidant pathways drives oxidative stress and encourages ferroptosis in susceptible cancer cells, forming a feedback loop [52, 53].

Ferroptosis is closely related to larger metabolic and signaling systems like amino acid metabolism, lipid biosynthesis, and oncogenic pathways. Specifically, important regulators like p53, NRF2, and AMP‐activated protein kinase (AMPK) control ferroptotic responses through redox status, iron homeostasis, and antioxidant defense. For example, p53 is effective (either promoting or inhibiting ferroptosis depending on the cellular context), whereas activation of NRF2 increases the antioxidant capability and provides protection against ferroptotic stress. Such a challenging regulatory environment emphasizes ferroptosis as a context‐dependent vulnerability in cancer and presents a great opportunity for targeted therapeutic action [18, 54].

## 3 Methods

In this review, recent advances in the investigation of the ferroptosis mechanism in aggressive cancers by natural products were systematically identified, analyzed, and summarized. To this end, an extensive search was performed on the Scopus and Web of Science online databases using the keywords “ferroptosis,” “aggressive cancers,” and “natural products.” The search was restricted to publications from 2021 to 2026, focusing on studies reporting relevant biological data published in high‐impact journals. It is important to note that no form of artificial intelligence (AI) was used in the drafting of this article. Gemini 3 (reasoning function) was used to generate Figure 2. However, the AI platforms QuillBot and Paperpal have been employed for AI detection and for proofreading and refining the text, respectively.

## 4 Natural Products Targeting Ferroptosis

Natural compounds have emerged as a rich and chemically diverse source of ferroptosis modulators, providing novel scaffolds that can target several points of ferroptotic regulation. More recently, several natural compounds have been reported as ferroptosis inducers, working according to the mechanisms of glutathione depletion, GPX4 inhibition, iron accumulation, and lipid peroxidation amplification. Notably, recent comprehensive analyses have identified over 150–170 natural products capable of modulating ferroptosis, highlighting the rapid expansion of this field [55, 56, 57, 58].

### 4.1 Direct and Indirect Inhibitors/Degraders of Glutathione Peroxidase 4

Glutathione peroxidase 4 is the fundamental enzymatic protective barrier against cellular lipoperoxidation, catalyzing the reduction of toxic lipid hydroperoxides to innocuous alcohols by the oxidation of glutathione. Therefore, GPX4 inactivation, through direct catalytic inhibition, transcriptional repression, or induction of its proteasomal degradation, appears to be one of the most efficient routes to induce ferroptosis. This subsection provides natural products whose central mechanism is based on the direct or indirect targeting of GPX4, which leads to the collapse of the antioxidant defense and the lethal accumulation of lipid ROS in aggressive and resistant tumor cell lines.

Selenomethionine (**1**) was previously revealed as a strong ferroptosis inhibitor acting through two mechanisms (Figure 5). From a biochemical system perspective, compound **1** acts as a selenium donor for the biosynthesis of GPX4 and initiates the transsulfuration pathway, which is associated with the induction of intracellular antioxidant capacity. The anti‐ferroptotic property of **1** remained remarkably potent after clustered regularly interspaced short palindromic repeats (CRISPR)‐induced suppression of GPX4, demonstrating another GPX4‐independent mechanism involving direct redox modulation by selenium‐mediated ROS scavenging. Experimental investigation showed that **1** protects against RSL3‐mediated ferroptosis in H1299 and A549 cancer cell lines and mouse embryonic fibroblast (MEF) cells. Of note, the protective effect was still present in MEF cells lacking the proper transsulfuration capacity, especially those that lack cystathionine β‐synthase, confirming the use of an alternative antioxidant mechanism. In vivo, treatment with **1** greatly alleviated the effects of cisplatin‐induced acute kidney injury by inhibiting ferroptosis‐associated destruction. These findings cumulatively ground **1** as a novel ferroptosis suppressor with a dual mechanism of bioactive action, encompassing the GPX4‐dependent enzymatic antioxidant defense as well as GPX4‐independent redox activity. Dual functionality offers novel perspectives to selenium‐assisted therapeutics aimed at ferroptosis‐mediated pathologies [59].

**FIGURE 5:**
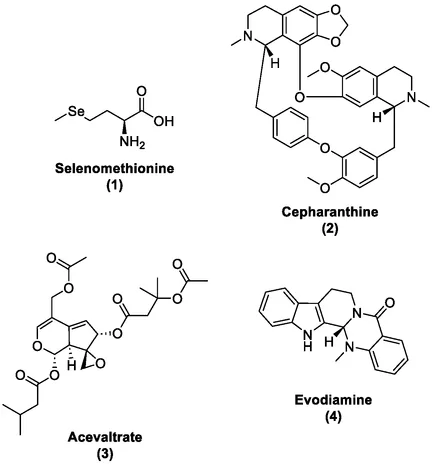
Natural products with anticancer activity, **1**–**4**.

Jing and collaborators investigated the plant‐derived natural product cepharanthine (**2**) as a ferroptosis inducer in gastric cancer (Figure 5). Though **2** is well established for anti‐inflammatory, immunomodulating, antiviral, antiparasitic, and antitumor actions, its mechanism of action in gastric cancer is not well established. Compound **2** was found to reduce the cell viability of MKN45 gastric cancer cells significantly (half maximal inhibitory concentration, IC_50_ = 12.28 μM) and promote ferroptosis through elevated intracellular Fe^2+^, ROS, and malondialdehyde (MDA) levels. On the other hand, a lesser degree of cytotoxicity was revealed in normal gastric epithelial cells (GES‐1; IC_50_ = 30.98 μM), proving some cancer selectivity. Determination of target specificities indicated that **2** binds directly to PTEN‐induced kinase 1 (PINK1), demonstrated through molecular docking, cellular thermal shift assay (CETSA), and drug affinity responsive target stability (DARTS) analysis. Treatment with compound **2** enhanced the thermal stability of PINK1, showing direct binding. Moreover, compound **2** also exhibits a multi‐target action involving runt‐related transcription factor 2 (RUNX2) and FUN14 domain‐containing protein 1 (FUNDC1). By stimulating PINK1‐mediated mitophagy, compound **2** promotes the FUNDC1‐dependent recruitment of GPX4 to mitochondria, resulting in its autophagic degradation. This loss of GPX4 weakens antioxidant defenses and induces ferroptosis. In vivo studies in a subcutaneous tumor model in athymic mice confirmed the antitumor activity of **2**, which is also associated with the induction of ferroptosis. Taken together, these results demonstrate that **2** is capable of exerting an antitumor effect through a multi‐target method involving mitophagy‐dependent ferroptosis [60].

In colorectal cancer, acevaltrate (**3**), a natural product, was found to be a novel ferroptosis inducer that targets both GPX4 and poly(rC)‐binding proteins 1/2 (PCBP1/2) (Figure 5). Ferroptosis was strongly and quickly induced by compound **3** in colorectal cancer cells, especially in RKO tumor cells. It was less toxic to normal intestinal epithelial cells but more effective against colorectal cancer cell lines (LoVo, SW620, HT29, RKO, and HCT116), with IC_50_ values ranging from 1.4 to 1.9 μM. Compound **3** dramatically caused G2/M phase cell cycle arrest in HCT116 and RKO cells, according to flow cytometry analysis. Furthermore, compound **3** significantly stopped oxaliplatin‐resistant HCT116 cells from proliferating. Mechanistically, compound **3** destabilized the antioxidant defense system by targeting GPX4, inhibiting its enzymatic activity and promoting its ubiquitin‐mediated degradation, in addition to increasing intracellular Fe^2+^ levels in colorectal cancer cells by reducing the expression of iron chaperones PCBP1/2. Compared to traditional ferroptosis inducers, this dual mechanism makes **3** much more effective. Mice subcutaneously inoculated with HCT116‐luc tumors and given oral corn oil or compound **3** once daily for 22 days showed significant reductions in tumor size, volume, and weight at doses of 25 and 50 mg·kg^–1^ when compared to the control group according to in vivo studies. Additionally, administration of **3** produced higher antitumor efficacy in athymic mice with RKO tumors than that in mice with HCT116 tumors, which is consistent with its cellular‐level activity. Notably, **3**’s therapeutic effect outperformed both first‐line clinical medications like capecitabine and TAS‐102 as well as established ferroptosis‐inducing agents. Crucially, compound **3** demonstrated better inhibitory effects in colorectal tumor organoids than those in traditional cell‐based models, highlighting its potential for chemical and translational development. This work is a groundbreaking attempt to find a small‐molecule inhibitor that targets GPX4 and PCBP1/2 at the same time, providing a new method for eradicating cancer cells via ferroptosis [61].

In a study by Li and colleagues, the natural product evodiamine (**4**) inhibits tripartite motif containing 26 (TRIM26)‐mediated stabilization of GPX4, causing ferroptosis in prostate cancer cells (Figure 5). When increasing concentrations of **4** were applied to prostate cancer cell lines (LNCaP, 22RV1, VCaP, PC‐3, and DU‐145), cellular activity was inhibited in a dose‐dependent manner. Prostate cancer cells’ viability, proliferation, invasion, and colony formation were all markedly inhibited by compound **4**. Interestingly, the apoptotic pathway was not involved in the cell death caused by **4** in PC‐3 and DU‐145 cells. On the other hand, compound **4** reduced GSH levels while raising total ROS production, lipid ROS levels, and MDA content, suggesting the induction of ferroptosis. The partial reversal of cell death following treatment with the ROS scavenger N‐acetylcysteine demonstrated the crucial role of ROS in **4**‐induced ferroptosis. **4** caused ferroptosis mechanistically by destabilizing the GPX4 protein in a TRIM26‐dependent manner. In vivo research utilizing a subcutaneous xenograft tumor model in athymic mice revealed that animals treated with compound **4** maintained their body weight in comparison to the control group. In comparison to the phosphate‐buffered saline (PBS) group, the compound **4**‐treated group showed a significant decrease in both tumor weight and volume. Compound **4**’s therapeutic potential was highlighted by its effective inhibition of tumor growth and induction of ferroptosis in tumor cells. Overall, this study shows that by preventing TRIM26‐mediated GPX4 stabilization and inducing ferroptosis, **4** has strong antitumor effects against prostate cancer. These results imply that compound **4**, a naturally occurring substance obtained from traditional Chinese medicine, is a viable treatment option for prostate cancer [62].

The natural flavonoid okanin (**5**), isolated from species such as *Bidens bipinnata* and *Coreopsis tinctoria*, has emerged as a promising antitumor agent in colorectal cancer (Figure 6). In HCT116 cells, compound **5** selectively inhibited cell proliferation while exhibiting low cytotoxicity toward nontumor cells, indicating a favorable therapeutic window. Mechanistically, **5** directly binds to peroxiredoxin‐5 (PRDX5), inhibiting its antioxidant activity and promoting intracellular ROS accumulation. This redox imbalance triggers cell death and is further amplified by the ubiquitin‐proteasome‐mediated degradation of both PRDX5 and GPX4. The concomitant depletion of these antioxidant defenses leads to the dual activation of apoptosis and ferroptosis. In vivo, **5** significantly suppressed tumor growth in xenograft models without detectable systemic toxicity, as evidenced by the absence of significant body weight loss, in contrast to 5‐fluorouracil. Collectively, these findings identify the PRDX5/GPX4 axis as a novel target of **5** and highlight its capacity to induce coordinated redox‐driven cell death. This study further underscores the potential of natural products as multifunctional scaffolds capable of modulating interconnected pathways, supporting the development of targeted and combinatorial therapeutic strategies for colorectal cancer [63].

**FIGURE 6:**
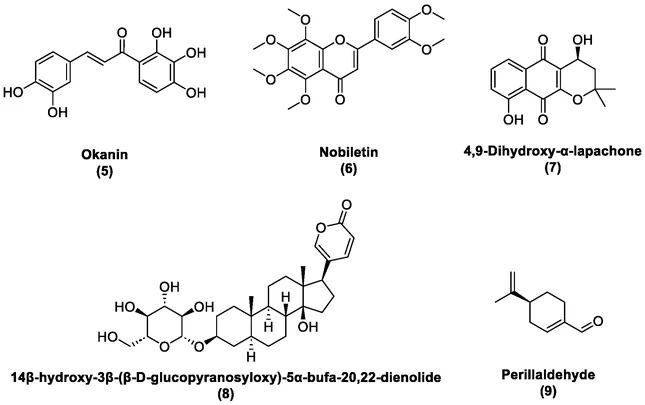
Natural products with anticancer activity, **5**–**9**.

Wu and colleagues investigated the polymethoxylated flavonoid nobiletin (**6**), isolated from citrus species such as *Citrus reticulata*, as a ferroptosis modulator in triple‐negative breast cancer (TNBC) (Figure 6). Compound **6** significantly inhibited cell proliferation and clonogenic capacity, indicating robust antitumor activity. Mechanistically, nobiletin induced ferroptosis by promoting ROS accumulation, increasing intracellular Fe^2+^ levels, elevating MDA, and depleting GSH, thereby establishing a pro‐oxidant environment. Target identification studies revealed that compound **6** directly binds to aldo‐keto reductase family 1 member C1 (AKR1C1), as confirmed by molecular docking, surface plasmon resonance, and CETSA. Functionally, the **6**–‍AKR1C1 interaction promotes ubiquitination and proteasomal degradation of GPX4, providing a direct mechanism for ferroptosis induction. This axis was validated by rescue experiments, in which ferroptosis inhibitors or ROS scavengers partially reversed the cytotoxic effects of nobiletin. Transcriptomic analysis further indicated that AKR1C1 regulation is associated with improved clinical outcomes, highlighting its potential translational relevance. In vivo, compound **6** effectively suppressed tumor growth and induced ferroptosis‐associated markers without detectable systemic toxicity. Collectively, these findings establish nobiletin as a natural scaffold that targets the AKR1C1/GPX4 axis to induce ferroptosis through a redox imbalance. This study highlights GPX4 ubiquitination as an emerging strategy and supports the potential of natural flavonoids in the development of ferroptosis‐based anticancer therapies [64].

He and coworkers reported a pharmacological study of 4,9‐‍dihydroxy‐α‐lapachone (**7**), a natural naphthoquinone derivative, with respect to its antitumor activity against TNBC (Figure 6). Cytotoxicity assays in TNBC cell lines (such as MDA‐MB‐231 and BT‐549) demonstrated significant antiproliferative activity of the compound, leading to strong cell death. The IC_50_ values of **7** are also 1.65 and 1.60 μg·mL^–1^ in 4T1 and MDA‐MB‐231 cells, respectively. Mechanistic studies revealed that **7** induces ferroptosis as a mode of cell death, evidenced by a marked increase in intracellular iron levels, increased MDA production, and significant lipid peroxidation. Molecular analysis suggested that ferroptosis induced by **7** is mediated by the breakdown of the GSH system and the subsequent downregulation of GPX4 expression. Moreover, the study indicates that **7** treatment modulates the metabolic landscape of TNBC cells by shifting the redox balance to oxidative stress. In vivo xenograft models demonstrated that **7** effectively suppressed tumor growth with a good safety profile. Taken together, these findings define **7** as a highly potent ferroptosis inducer and a promising lead for the development of therapeutics against aggressive TNBC subtypes [65].

Liu and coworkers studied the anticancer efficacy of a natural product, 14β‐hydroxy‐3β‐(β‐*D*‐glucopyranosyloxy)‐5α‐‍bufa‐20,22‐dienolide (**8**), isolated from *Helleborus thibetanus* Franch, in TNBC (Figure 6). The cytotoxicity assays in different TNBC cell lines showed potent antiproliferative activity, significantly inhibiting the cell growth and migration ability. Mechanistic studies showed that **8** exerts its effects through modulation of the FOS‐like 2, AP‐1 transcription factor subunit/forkhead box C1 (FOSL2/FOXC1) signaling axis. It was found that the compound suppressed the expression of FOSL2 and subsequently suppressed the downstream transcription factor FOXC1. This signaling downregulation causes ferroptosis in TNBC cells, which is characterized by a dramatic increase in lipid peroxidation, increased accumulation of ROS, and decreased expression of the critical antioxidant protein GPX4. These findings suggest that the FOSL2/FOXC1 axis serves as a critical regulatory node of TNBC ferroptosis sensitivity. In summary, this study demonstrates that **8** is a potent ferroptosis‐inducing agent and underscores the potential of targeting the FOSL2/FOXC1 pathway in the clinical management of aggressive TNBC [66].

Wang and collaborators described the pharmacological study of perillaldehyde (**9**), a monocyclic terpenoid from Perilla frutescens, in which the antitumor effect of **9** was evaluated in gastric cancer (Figure 6). In vitro assays showed significant inhibition of proliferation and migration in AGS, HGC27, and MFC cell lines, which was corroborated by in vivo xenograft models in BALB/c nude mice. Mechanistic studies showed that **9** induced ferroptosis, which was evidenced by decreased mitochondrial membrane potential, increased ROS accumulation, and increased intracellular Fe^2+^ and lipid peroxidation levels. Molecular analysis indicated that **9** inhibited the system X_c_^−^/GSH/GPX4 axis and damaged antioxidant defense. In addition, the study showed that **9** regulates ferroptosis through the P62‐Keap1‐NRF2 signaling pathway that seems to serve as a compensatory stress‐adaptive mechanism. Interestingly, coadministration experiments showed a synergistic effect of **9** on the ferroptotic effect of RSL3 as well as conventional chemotherapeutics, such as doxorubicin and cisplatin. Collectively, these findings suggest that **9** is a multimodal ferroptosis inducer in gastric cancer. Our findings support the idea that **9**’s can be a therapeutic scaffold for the development of ferroptosis‐sensitizing strategies to improve the outcome of aggressive gastric cancer [67].

Chen and coworkers reported the investigation of undecane‐2‐‍one (**10**), a natural compound that was tested for its antitumor efficacy in renal cell carcinoma and its potential to modulate sensitivity to sunitinib (Figure 7). In vitro assays showed that **10** could significantly inhibit the viability, proliferation, and migration of renal cell carcinoma cells in a concentration‐dependent manner. Mechanistic studies suggest that **10** induces ferroptosis, as evidenced by the accumulation of intracellular Fe^2+^, the increased production of ROS, and the enhanced lipid peroxidation. Molecular studies identify the signal transducer and activator of transcription 3, STAT3/GPX4 signaling axis as a potential target, and computational docking analysis and immunoblotting show that **10** binds to STAT3 stably, leading to downregulation of p‐STAT3 and subsequent inhibition of GPX4. Additionally, **10**, in addition to sunitinib, was shown to increase therapeutic sensitivity in renal cell carcinoma cells, with a decreased IC_50_ value and more marked inhibition of tumor progression than monotherapy. The study indicates that 50 μM of **10** significantly sensitizes renal cell carcinoma cells to sunitinib. This synergistic effect is demonstrated by the significant reduction of IC_50_ values of sunitinib from 11.77 to 7.404 μM in 786‐O cells and from 11.36 to 7.231 μM in A498 cells, thus enhancing the therapeutic efficacy of the drug. In conclusion, these data imply that **10** acts as a chemosensitizing agent in renal cell carcinoma through inhibition of the STAT3/GPX4 axis and consequently promotes ferroptotic cell death. The study points to **10** as a promising candidate for overcoming drug resistance in renal cell carcinoma therapy [68].

**FIGURE 7:**
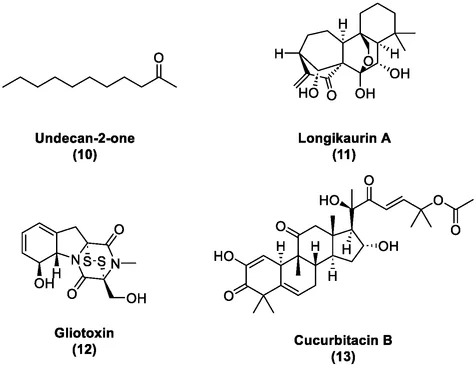
Natural products with anticancer activity, **10**–**13**.

Meng and collaborators reported the evaluation of the natural diterpenoid longikaurin A (**11**) isolated from *Isodon ternifolius* for antitumor activity against glioblastoma (Figure 7). In vitro assays demonstrated that **11** strongly inhibited the proliferation, migration, and invasion of glioblastoma cell lines with potent IC_50_ values of 1.48 μM (U87) and 1.62 μM (U251) at 48 h. Mechanistic studies indicated that **11** induced ferroptosis, as evidenced by a significant increase in ROS production, lipid peroxidation, and downregulation of GPX4. The study showed that the action of **11** is through an epigenetic mechanism, causing methylation of the promoter of the GPX4 gene, leading to transcriptional silencing of this protective enzyme. In vivo experiments in xenograft mouse models indicated that treatment with **11** significantly reduced the tumor volume, and this effect was reversed by the ferroptosis inhibitor, ferrostatin‐1. Together, these findings indicate that **11** is a potent ferroptosis inducer in glioblastoma and point to the epigenetic regulation of GPX4 as a promising therapeutic target for overcoming resistance to conventional glioblastoma treatments [69].

Chen and coworkers thoroughly investigated the antitumor efficacy of gliotoxin (**12**), a natural mycotoxin produced by *Aspergillus fumigatus*, with special emphasis on its potential to induce ferroptotic cell death (Figure 7). In vitro, gliotoxin showed potent cytotoxic activity with IC_50_ values of 0.24 μM (H1975 cells) and 0.45 μM (MCF‐7 cells) after 72 h of treatment. Mechanistic studies showed that **12** acts as a ferroptosis inducer by effectively downregulating GPX4 expression to abolish cellular antioxidant defenses, resulting in fatal accumulation of iron‐dependent lipid peroxides. These results support the notion that **12** can act as a pro‐ferroptotic scaffold and strengthen the idea that targeting vulnerabilities related to ferroptosis may represent a viable therapeutic strategy to overcome acquired drug resistance in multiple oncological malignancies [70].

Huang and colleagues reported biological evaluation of cucurbitacin B (**13**), a naturally occurring triterpenoid, which displayed potent antitumor activity in human nasopharyngeal carcinoma cells (Figure 7). Cytotoxicity assays revealed that **13** possesses excellent antiproliferative activity against these malignant cells. Mechanistic studies confirmed the characteristic morphological changes of the mitochondrial ultrastructure in ferroptosis through transmission electron microscopy. Moreover, **13** treatment resulted in a significant intracellular accumulation of ferrous iron Fe^2+^ and depletion of GSH, which are critical events for the destabilization of cellular redox homeostasis. Molecular analysis revealed that **13** induced a multifaceted ferroptosis cell death program through extensive lipid peroxidation and the suppression of GPX4. In addition to ferroptosis, the compound demonstrated other antitumor effects, such as inhibition of microtubule polymerization, cell cycle arrest, and suppression of migratory and invasive capabilities. Collectively, these findings establish **13** as a potent inducer of ferroptosis, highlighting its therapeutic relevance in the treatment of nasopharyngeal carcinoma and expanding the understanding of its antitumor mechanisms beyond classical apoptotic pathways [71].

### 4.2 Modulators of System X_c_^–^ (SLC7A11) and Cystine Transport

The cystine/glutamate antiporter X_c_^–^ system, containing its functional subunit SLC7A11 (xCT), is required for cellular glutathione (GSH) synthesis. Natural products that specifically modulate this subunit block cystine uptake, resulting in rapid depletion of GSH stores and triggering ferroptosis by collapse of tumor redox control.

Recently, macelignan (**14**) has been found to be a natural product used to induce ferroptosis in macrophages by targeting the cystine transporter SLC7A11 (Figure 8). In hepatocellular carcinoma (HCC)‐conditioned macrophages, compound **14** induced depletion of GSH, increased lipid peroxidation, and downregulated SLC7A11 and GPX4, suggesting activation of ferroptosis. On the basis of the mechanism, **14**‐mediated ferroptosis induction suppresses M2 macrophage polarization by blocking the STAT6/PPAR‐γ/KLF4 signaling pathway. This reprogramming disturbed the C–C motif chemokine ligand 2 (CCL2)‐mediated crosstalk between tumor‐associated macrophages and tumor cells in the HCC microenvironment, resulting in the suppression of macrophage‐induced tumor growth, migration, and epithelial–mesenchymal transition. Molecular docking studies suggested that **14** interacts with the SLC7A11 substrate‐binding site and is responsible for critical binding to the residues Lys198, Thr56, and Ala60, with a predicted binding energy of −8.4 kcal·mol^–1^. Consistently, overexpression of SLC7A11 reversed the immunomodulatory and ferroptosis‐inducing effects of compound **14**, verifying its active character. Compound **14** effectively suppressed tumor growth and metastatic dissemination in xenograft models with no evidence of any systemic toxic effects in vivo. These results jointly illustrate that natural product‐induced ferroptosis may reprogram tumor‐associated macrophages and alter tumor‐promoting signaling pathways in a manner under‐researched in the cancer literature [72].

**FIGURE 8:**
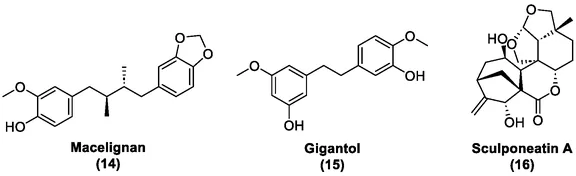
Natural products with anticancer activity, **14**–**16**.

Chen and coworkers reported the systematic study of the antitumor efficacy of the naturally occurring dibenzyl compound gigantol (**15**), isolated from *Dendrobium orchid* species, in lung cancer (Figure 8). Compound **15** was shown to potently inhibit tumor growth by inducing ferroptotic cell death in both in vitro and in vivo models. Mechanistic studies identified SLC7A11, a key subunit of the cystine/glutamate antiporter system (X_c_^–^), as a direct molecular target. **15** inhibits SLC7A11, thus restricting the uptake of extracellular cystine, leading to decreased GSH synthesis and disruption of cellular redox homeostasis. This is followed by the depletion of GSH, inactivating the antioxidant enzyme GPX4, which promotes iron‐dependent oxidative stress and lipid peroxidation. These results suggest that **15** is a potent inducer of ferroptosis and that the SLC7A11‐GPX4 axis is a promising therapeutic target for reverting tumor progression in lung cancer [73].

Sculponeatin A (**16**), an ent‐kaurane diterpenoid isolated from *Isodon sculponeatus*, was biologically evaluated by Peng and collaborators, who identified it as a potent inducer of ferroptosis in breast cancer (Figure 8). Cytotoxicity assays in human breast cancer cell lines (MCF‐7, MDA‐MB‐231, and BT‐474) demonstrated 24 h IC_50_ values of 60.68, 55.69, and 53.50 nM, respectively, and significantly lower toxicity toward the normal mammary epithelial cell line MCF‐10A (IC_50_ = 189.30 nM). Mechanistic studies showed that **16** induced ferroptosis, as demonstrated by the loss of mitochondrial membrane potential, intracellular Fe^2+^ accumulation, increased ROS and MDA production, and GSH depletion. This effect was associated with downregulation of the subunit of the cystine/glutamate antiporter xCT (SLC7A11), resulting in impaired antioxidant defenses. Comprehensive molecular analysis revealed that **16** directly bound to the ETS domain of the E26 transformation specificity‐1 (ETS1), thereby inhibiting xCT transcription by disrupting the interaction of ETS1 with the xCT promoter. Moreover, **16** seems to act as an enhancer of protein–protein interactions by favoring the interaction of ETS1 with the E3 ubiquitin ligase synoviolin 1 (SYVN1), which mediates the specific ubiquitination of ETS1 at the K318 site and its subsequent proteasomal degradation. In vivo experiments using an MDA‐MB‐231 xenograft mouse model showed that **16** significantly inhibits tumor growth at doses of 1 and 3 mg·kg^–1^ with no apparent systemic toxicity. These findings demonstrate that **16** induces xCT‐dependent ferroptosis through SYVN1‐mediated ETS1 degradation, which highlights this natural diterpenoid as a promising scaffold for breast cancer therapy and the design of ETS1‐targeted protein degraders [74].

### 4.3 Dual Modulators of the System X_c_^–^/GPX4 Axis

A highly effective strategy to induce ferroptotic cell death involves co‐targeting the SLC7A11 and GPX4, which blocks both the import of a precursor for glutathione biosynthesis and the enzymatic barrier to lipid hydroperoxidation. Natural products that act as dual modulators synergistically target the major antioxidant mechanisms of tumor cells, often by targeting upstream regulatory networks, such as the p53 axis. This subsection presents natural compounds that inhibit SLC7A11 activity but induce GPX4 inactivation or degradation, leading to the catastrophic disruption of redox homeostasis and the potent induction of ferroptosis in resistant cancer models.

Luo and collaborators demonstrated the potent antiproliferative effects of the natural product rottlerin (**17**) in HCC by causing ferroptosis (Figure 9). This effect revealed enhanced lipid peroxidation and ferroptotic cell death that were reversed by the ferroptosis inhibitor, ferrostatin‐1. Compound **17** was assayed in HLE and LM3 cell lines with IC_50_ values of 2.25 and 0.94 μM, respectively. In contrast, a larger IC_50_ value (4.59 μM) was detected in normal LX‐2 hepatic cells, implying, to some extent, cancer‐targeted cytotoxicity. At subtherapeutic amounts, **17** markedly upregulated the efficacious activity of traditional ferroptosis inducers RSL3 and sorafenib. Importantly, this chemosensitizing process survived the loss of protein kinase C δ (PKCδ), suggesting that the ferroptotic activity of **17** is not dependent on its reported PKCδ inhibitory function. Compound **17** is involved in the ubiquitin–proteasome‐mediated degradation of two of the major drivers of ferroptosis resistance, cystine transporter SLC7A11 and GPX4, causing antioxidant defenses to be weakened and lipid peroxidation to be accelerated. This phenotypic double‐target mode of the degradation approach differentiates rottlerin from conventional single‐target phytochemicals and provides the basis of its vigorous ferroptosis‐promoting properties. In vivo assays in animals, where it was shown in HLE xenograft mouse models, showed that **17** significantly suppressed tumor growth, with enhanced efficacy observed in combination with sorafenib. This combination treatment led to increased levels of MDA, a lipid peroxidation marker, compared to those with sorafenib only. Western blot analysis of the tumor tissue consistently disclosed significant downregulation of SLC7A11 and GPX4, and the combination treatment group had the lowest expression levels. This confirmed the value of **17** as a ferroptosis‐inducing agent and likely for combination therapy in HCC [75].

**FIGURE 9:**
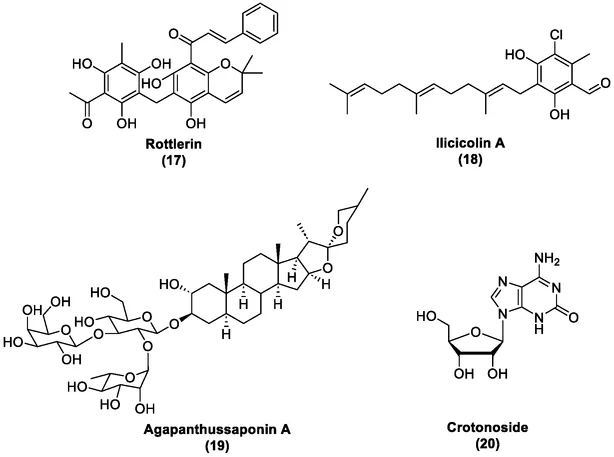
Natural products with anticancer activity, **17**–**20**.

Li and coworkers investigated the role of ilicolin A (**18**) in overcoming enzalutamide resistance in castration‐resistant prostate cancer (CRPC) (Figure 9). Using C4‐2B and 22Rv1 prostate cancer cell lines, the authors demonstrated that overexpression of the RAR‐related orphan receptor C (RORC) significantly enhanced cell proliferation and upregulated ferroptosis‐associated proteins, including ferritin heavy chain 1 (FTH1), GPX4, and SLC7A11. Molecular docking studies indicated that ROR‐γ exhibited binding affinity toward FTH1, GPX4, and SLC7A11, with binding energies of −24.3, −11.1, and −1.3 kcal·mol^–1^, respectively, suggesting a regulatory role in ferroptosis‐related pathways. Functional assays further revealed that RORC overexpression markedly increased the resistance to enzalutamide. Specifically, the IC_50_ value increased from 19.38 μM in parental C4‐2B cells to 518.7 μM in RORC‐overexpressing cells (*oeRORC*/C4‐2B), corresponding to a resistance index of >15. This finding was corroborated by an independently established enzalutamide‐resistant cell line (C4‐2B/ENZR), which exhibited an IC_50_ value of 605.9 μM. Treatment with **18** significantly suppressed CRPC cell proliferation by downregulating RORC expression and restoring ferroptotic sensitivity. Mechanistically, compound **18** increased intracellular Fe^2+^ levels, ROS accumulation, and MDA production while reducing GSH levels, collectively promoting ferroptosis. In addition, **18** enhanced the interaction between ROR‐γ and GPX4, further implicating this axis in ferroptosis regulation. Western blot analysis confirmed that treatment with compound **18** reduced the expression of AR and its splice variant AR‐V7, key drivers of CRPC progression and therapeutic resistance. In vivo studies demonstrated that tumors derived from RORC‐overexpressing cells exhibited accelerated growth compared to that of control tumors. Notably, **18** significantly inhibited tumor growth, with efficacy comparable to that of erastin (positive control) and showed enhanced antitumor activity when combined with enzalutamide. Histological evaluation revealed no significant tissue toxicity, supporting the favorable safety profile of compound **18**. Collectively, these findings identify RORC as a critical regulator of ferroptosis and enzalutamide resistance and highlight **18** as a promising natural product capable of restoring ferroptotic vulnerability in CRPC [76].

Iguchi and coworkers reported the phytochemical investigation of the subterranean parts of *Agapanthus africanus*, leading to the isolation of three spirostan‐type steroidal glycosides, among which agapanthussaponin A (**19**) was identified as the most active compound (Figure 9). Cytotoxicity assays in small‐cell lung carcinoma (SCLC; SBC‐3 cells) revealed IC_50_ values of 0.56, 1.4, and 7.4 μM for compounds **19** and its analogs, respectively, indicating a structure–activity relationship associated with the degree of unsaturation in the steroidal scaffold. Mechanistic studies demonstrated that compound **19** induces G2/M cell cycle arrest and activates intrinsic mitochondrial apoptosis, as evidenced by the loss of mitochondrial membrane potential, increased ROS production, and activation of caspases‐3 and ‐9. In parallel, agapanthussaponin A also triggered ferroptosis, characterized by intracellular Fe^2+^ accumulation, increased lipid peroxidation, and elevated MDA levels. This effect was associated with upregulation of transferrin and its receptor (CD71), promoting enhanced iron uptake, along with inhibition of the SLC7A11/GPX4 axis, leading to impaired glutathione synthesis and reduced detoxification of lipid peroxides. Additionally, activation of the NRF2/HO‐1 pathway suggests a stress‐adaptive response that may contribute to increased intracellular iron availability. Collectively, these findings establish compound **19** as a multimodal inducer of cell death, integrating apoptosis and ferroptosis through the coordinated disruption of redox homeostasis and iron metabolism. This study highlights steroidal saponins as promising natural scaffolds for targeting ferroptotic vulnerabilities in aggressive cancers such as SCLC [77].

Ma and coworkers conducted a pharmacological study of crotonoside (**20**), a guanosine analog derived from *Croton tiglium* L., examining its effects on mitochondrial dysfunction and ferroptosis in acute myeloid leukemia (AML) cells (Figure 9). Cytotoxicity tests indicated that there is antiproliferative activity, yielding IC_50_ values of 6.16 μM against MOLM‐13 cells and 8.99 μM against KG‐1a cells. According to mechanistic evaluations, **20** disrupts mitochondrial respiration, resulting in a marked decrease in both basal and maximal respiratory capacities. This dysfunction is characterized by a dose‐dependent decrease in the mitochondrial membrane potential, accompanied by mitochondrial swelling and a marked increase in intracellular ROS. Molecular docking studies indicate that this mitochondrial dysfunction might occur via the binding and inhibition of mitochondrial DNA polymerase γ. Concurrently, **20** induced ferroptosis, as evidenced by heightened intracellular Fe^2+^ levels, increased MDA accumulation, and a significantly decreased GSH/GSSG ratio. The ferroptotic effect was linked to a decrease in the system X_c_^−^/GPX4 axis (SLC7A11 and GPX4) and an increase in ACSL4, which facilitates lipid peroxidation. Moreover, **20** regulated autophagy through the downregulation of the p62/KEAP1 signaling pathway, indicating that increased autophagic activity could lead to the degradation of antioxidant enzymes, thereby increasing sensitivity to ferroptosis. Overall, these results indicate that **20** functions as a multimodal inducer of cell death in AML cells by combining mitochondrial DNA damage with autophagy‐driven ferroptosis. According to this study, targeting mitochondrial integrity and exploiting ferroptotic vulnerabilities could serve as a promising therapeutic approach for AML [78].

Yu and collaborators conducted a pharmacological study on erianin (**21**), a natural compound obtained from *Dendrobium officinale*, and assessed its anticancer properties in the context of osteosarcoma (Figure 10). Human MG‐63 and U‐2OS cell lines were subjected to in vitro cell viability assays, which showed remarkable antiproliferative activity at IC_50_ values of 1.22 and 1.48 μM, respectively. Additional phenotypic assays indicated that **21** significantly inhibits colony formation and cell migration. According to mechanistic studies, **21** triggers apoptosis and cell cycle arrest, all the while activating the ferroptotic pathway. This effect was marked by reduced expression of GPX4 and the SLC7A11 transporter, which compromised antioxidant defenses, resultingin increased intracellular ROS levels and raised MDA levels, a sign of significant lipid peroxidation. Experiments in vivo utilizing nude mouse xenograft models demonstrated that treatment with **21** notably decreases both the volume and weight of tumors, all while showing no clear signs of systemic toxicity. Taken together, these results indicate that **21** is a multimodal anticancer agent that combines the initiation of apoptosis with the triggering of ferroptosis by disturbing the redox balance. These findings suggest that **21** may serve as a valuable framework for the creation of therapeutic strategies aimed at exploiting ferroptotic weaknesses in osteosarcoma treatment [79].

**FIGURE 10:**
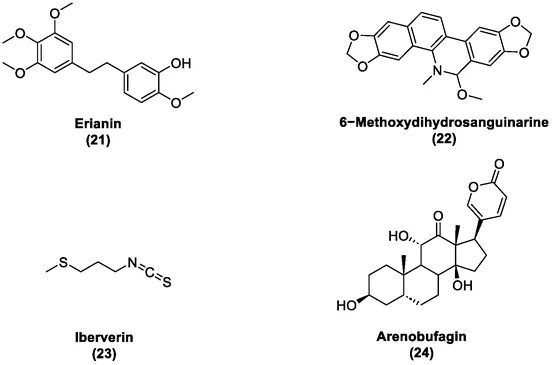
Natural products with anticancer activity, **21**–**24**.

From research led by Han and coworkers, the compound 6‐methoxydihydrosanguinarine (**22**) was studied for its effects on liver cancer cells (Figure 10). Instead of normal growth, treated HepG2 and Huh7 cells showed reduced survival rates, measuring 6.32 and 7.54 μM in potency tests. Death among these cells appears tied to iron‐driven processes, supported by a higher Fe^2+^ presence inside them. Alongside this shift came increases in ROS and MDA, markers linked to oxidative damage. Notably, protective proteins like GPX4 and SLC7A11 dropped during exposure, weakening the cells’ ability to manage oxidation. As an outcome, the balance within their internal environment became unstable. In addition, activity in the PI3K/AKT/mTOR network decreased, possibly making conditions worse for cell recovery under stress. These results, taken together, position **22** as an effective trigger of ferroptosis within HCC cell contexts. Natural alkaloids gain relevance here—not only through their influence on metabolic disruption but also via modulation of oxidative balance, wherein ferroptosis emerges as a central mechanism worth investigating in difficult‐to‐treat liver cancers [80].

Yang and colleagues reported the investigation of iberverin (**23**), a natural organosulfur compound, for its antitumor efficacy in HCC (Figure 10). The in vitro assays showed that **23** has a potent cytotoxic activity against HCC cells and has an IC_50_value of 14.83 μM (HepG2) and 19.74 μM (Huh7), respectively, after 48 h of treatment, indicating significant inhibitory potential on cell proliferation. Mechanistic studies suggest that **23** induces ferroptosis, as shown by accumulation of intracellular Fe^2+^, increased production of ROS, and elevated lipid peroxidation, which were reversed by ferroptosis inhibitor ferrostatin‐1. Molecular data show that **23** treatment induces the downregulation of important protective proteins, GPX4 and SLC7A11, leading to the disturbance of the cellular antioxidant defense system. Additionally, in vivo xenograft mouse models confirmed the effective inhibition of tumor growth by **23** administration without detectable systemic toxicity. In combination, these results indicate that **23** is a potent inducer of ferroptotic cell death in HCC and identify the SLC7A11/GPX4 axis as a potential therapeutic target. The present study identifies **23** as a promising natural scaffold for the development of targeted therapies against HCC by exploiting iron‐dependent oxidative stress [81].

Long and colleagues investigated the antitumor efficacy of arenobufagin (**24**), a natural bufadienolide isolated from toad venom, on gastric cancer (Figure 10). Compound **24** showed potent cytotoxicity in vitro, with IC_50_ values of 140 and 260 nM against MGC‐803 and SGC‐7901 cells, respectively. Mechanistic studies showed that **24** induced ferroptosis, evidenced by the accumulation of intracellular iron, increased ROS levels, and increased lipid peroxidation. Molecular studies showed that the NRF2/SLC7A11/GPX4 signaling pathway was the main target of **24**. Compound **24** caused repression of NRF2, which led to the downregulation of its downstream targets SLC7A11 and GPX4 and impaired the cellular antioxidant defense. These results were further confirmed by overexpression studies, in which high expression of NRF2 could effectively reverse **24**‐induced ferroptosis. Furthermore, in vivo xenograft models confirmed that **24** treatment significantly decreased tumor growth. This study collectively establishes **24** as a potent pro‐ferroptotic agent in gastric cancer and highlights the NRF2/SLC7A11/GPX4 axis as a viable therapeutic target [82].

Zhao and coworkers reported the systematic investigation of the natural naphthoquinone, β‐lapachone (**25**), for its antitumor efficacy in colorectal cancer (Figure 11). In vitro assays revealed that this compound showed potent cytotoxic effects against colorectal cancer cells with IC_50_ values of 2.397 μM (SW620), 2.946 μM (SW480), and 2.036 μM (DLD‐1) after 24 h treatment. Mechanistic studies showed that **25** induced nuclear receptor coactivator 4 (NCOA4)‐dependent ferritinophagy and autophagic degradation of ferritin, resulting in an increase in the labile iron pool. This process is dependent on the activation of the C‐Jun N‐terminal kinase (JNK) signaling pathway, resulting in the downregulation of critical antioxidant proteins, SLC7A11 and GPX4, thus promoting iron‐dependent oxidative stress and ferroptosis. These results were corroborated in colorectal cancer cell lines and xenograft models and demonstrated that **25** effectively disrupts iron homeostasis. Overall, the present work highlights the potent pro‐ferroptotic activity of **25** in colorectal cancer and unveils the JNK/NCOA4/ferritinophagy axis as a novel and viable therapeutic target for colorectal cancer management [83].

**FIGURE 11:**
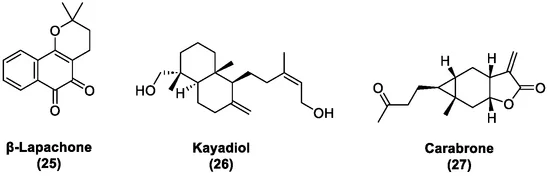
Natural products with anticancer activity, **25**–**27**.

He and coworkers reported the biological evaluation of kayadiol (**26**), a diterpenoid isolated from *Isodon rubescens*, showing its antitumor effect in extranodal natural killer/T‐cell lymphoma (NKTCL) cell lines (Figure 11). Cell viability assays in the YT cell line and peripheral lymphocytes from patients demonstrated that **26** inhibited cell growth in a dose‐dependent manner, with IC_50_ values significantly lower than in normal cells, suggesting a promising therapeutic selectivity. Mechanistic studies confirmed that **26** induces ferroptosis, as shown by reversal of cell death following treatment with specific ferroptosis inhibitors such as ferrostatin‐1, replenishment of GSH levels, and reduction of ROS by antioxidants. Proteomic analysis and molecular assays demonstrated that **26** activates the p53 protein, which in turn represses the expression of SLC7A11 and GPX4, key elements of the cellular antioxidant system, leading to the accumulation of lipid peroxidation and the failure of redox homeostasis. Gene silencing experiments (siRNA and CRISPR/Cas9) confirmed that p53 is a key causal mediator, since p53 deficiency significantly alleviated the effect of **26**. Moreover, the treatment showed a synergistic effect with L‐asparaginase or cisplatin to enhance cytotoxicity in NKTCL cells. Collectively, these findings demonstrate that **26** induces p53‐mediated ferroptosis, and the p53/SLC7A11/GPX4 axis may be a potential therapeutic target to overcome resistance and aggressiveness in NKTCL [84].

Zheng and coworkers reported the biological evaluation of carabrone (**27**), a carabrane‐type sesquiterpenolide isolated from *Carpesium cernuum*, and identified its potential as an inducer of ferroptosis in pancreatic cancer (Figure 11). Cytotoxicity assays in the human pancreatic cancer cell line SW1990 showed that **27** inhibited cell viability in a dose‐ and time‐dependent manner. Mechanistic studies showed that **27** induced ferroptosis, as evidenced by a significant increase of intracellular ROS and lipid peroxidation and depletion of intracellular GSH. In addition, the iron chelator deferoxamine (DFO) and lipophilic antioxidant ferrostatin‐1 successfully rescued the cell viability inhibition, confirming the iron‐dependent oxidative nature of cell death. Molecular analyses suggest that **27** modulates the expression of essential ferroptosis‐related proteins, importantly downregulating GPX4 and the system X_c_^–^ subunit SLC7A11, thereby compromising cell antioxidant defense systems. Collectively, these findings define **27** as a potent natural scaffold for the targeting of pancreatic cancer cells and underscore the potential of ferroptosis induction as a therapeutic strategy against this highly resistant malignancy [85].

### 4.4 Regulators of Iron Homeostasis, Ferritinophagy, and Labile Iron Pool

Intracellular iron overload is the metabolic engine of ferroptosis that fuels the Fenton reaction for ROS that cause lipid peroxidation. Cellular sensitivity to ferroptosis is tightly associated with iron availability, which is controlled by iron import (TFR1), reduction (STEAP3), storage (FTH1), and selective autophagic degradation of ferritin (ferritinophagy mediated by NCOA4 and LC3B). Here, we highlight natural products that modulate iron transport, storage, or autophagic turnover to increase the labile iron pool, thereby lowering the threshold for lipid ROS execution and sensitization of malignant cells.

Wang and collaborators reported the pharmacological evaluation of a natural structural analog of capsaicin, homocapsaicin II (**28**) extracted from *Capsicum annuum* L., on antitumor activity against colorectal cancer (Figure 12). In vitro and in vivo assays revealed that **28** induced potent antiproliferative effects via the induction of ferroptotic cell death. Mechanistic studies showed that **28** directly binds to kinesin family member 11 (KIF11) and stabilizes the protein by inhibiting its ubiquitin‐proteasome‐mediated degradation. The resultant KIF11 accumulation promotes cholesterol biosynthesis that facilitates centrosome amplification (CA). This process, together with KIF11‐dependent defects in centrosome separation, leads to multipolar mitosis. Our study shows that the combination of cholesterol‐induced CA and KIF11‐mediated multipolarity is a key inducer of ferroptosis, indicated by the increase in intracellular Fe^2+^ levels, augmented lipid peroxidation, the decrease in the GSH/GSSG ratio, and the regulation of ferroptosis‐related markers (ACSL5 and STEAP3 up‐regulation; FTH1 and GPX4 down‐regulation). Altogether, these results define **28** as a novel dual‐pathway inducer of ferroptosis connecting metabolic dysregulation to mitotic catastrophe. They demonstrate that the KIF11/cholesterol/centrosome axis represents a key regulatory node and position **28** as a promising therapeutic scaffold to target metabolic‐mitotic vulnerabilities in colorectal cancer [86].

**FIGURE 12:**
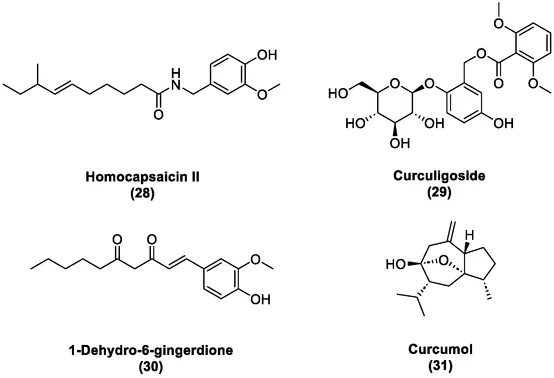
Natural products with anticancer activity, **28**–**31**.

Ma and colleagues reported a phytochemical investigation of curculigoside (**29**), a phenolic compound isolated from the rhizome of *Curculigo orchioides* Gaertn, and evaluated its antitumor efficacy in osteosarcoma (Figure 12). In vitro studies showed that **29** could markedly inhibit osteosarcoma cells’ proliferation and induce cell death in a dose‐ and time‐dependent manner. Mechanistic studies show that **29** modulates the tumor microenvironment by inducing differentiation of RAW264.7 cells into M1‐type macrophages, which is characterized by upregulation of CD86 and downregulation of CD206 and is mediated by activation of the NF‐κB pathway and subsequent upregulation of inducible nitric oxide synthase (iNOS). Compound **29**‐treated macrophage‐conditioned medium (CM) was proven to inhibit osteosarcoma cell viability and induce ferroptosis, as indicated by the accumulation of intracellular Fe^2+^, increased ROS production, and increased levels of lipid peroxidation markers, including MDA and 4‐hydroxynonenal (4‐HNE). Furthermore, CM treatment led to the degradation of GPX4 and dysregulation of iron metabolism‐associated proteins such as TFR1 and FTH1, which was mitigated by iron‐chelating agents DFO and Fer‐1. In vivo, **29** treatment significantly suppressed tumor growth in mouse xenograft and patient‐derived mini‐PDX models. Immunohistochemical analysis confirmed a shift in macrophage polarization toward the M1 phenotype and the induction of ferroptotic markers in the tumor tissue. Collectively, these results indicate the multimodal therapeutic role of curculigoside in osteosarcoma and the immune‐modulatory activation of ferroptotic cell death through iron‐dependent oxidative stress. **29** may be a potential candidate to improve the efficacy of current chemotherapy regimens in osteosarcoma [87].

Tran and colleagues reported the evaluation of a ginger‐derived compound, 1‐dehydro‐6‐gingerdione (**30**), for its anticancer potential against human breast cancer (MDA‐MB‐231) cells (Figure 12). MTT and colony formation assays revealed that **30** has significant cytotoxic effects with an IC_50_ of 71.13 μM, suggesting a dose‐dependent inhibition of cell proliferation. Proteomics analysis, validated by qRT‐PCR and immunoblotting, demonstrated the induction of the ferroptosis pathway by **30**, as shown by a significant increase in ROS production, lipid peroxidation (MDA levels), and intracellular Fe^2+^ accumulation. Mechanistic studies showed that treatment led to up‐regulation of pro‐ferroptotic proteins including HO‐1, autophagy‐related 7 (ATG7), and microtubule‐associated protein 1 light chain 3 Beta (LC3B), downregulation of FTH1 expression, and evidence of dysfunction of the mitochondria. These findings are consistent with in vivo data from a xenograft mouse model showing that **30** treatment reduced tumor volume without systemic toxicity. Moreover, the in silico modeling showed a stable interaction of **30** with the key proteins involved in the ferroptotic process. Together, these results show that **30** is a strong inducer of ferroptosis in breast cancer cells, indicating that the compound could be a potential therapeutic strategy for breast cancer management by targeting iron metabolism and redox homeostasis [88].

Zhao and collaborators studied the antitumor potential of curcumol (**31**), a sesquiterpene natural product, against colon cancer (Figure 12). GPX4 is significantly upregulated in colon cancer tissues, and its high expression correlates with poor patient prognosis, identifying it as a critical molecular target, according to the researchers. **31** in vitro suppresses the proliferation of the human colon cancer cell line SW480 in a dose‐dependent manner, with an IC_50_ value of 55.61 μM, and induces ferroptosis. The mechanism of **31**‐induced ferroptosis is to promote iron Fe^2+^ overload, increase the levels of ROS and MDA, and decrease the levels of superoxide dismutase 1 (SOD1). Molecular docking simulation and co‐immunoprecipitation assay reveal that **31** directly binds to GPX4, resulting in ubiquitination and degradation of GPX4, and then modulates the expression of ferroptosis‐related proteins such as FTH1 and TFR1. In vivo studies further confirm that **31** inhibits tumor growth in mouse models. Collectively, these findings suggest that **31** exerts anti‐colon cancer effects by targeting GPX4 degradation to induce ferroptotic cell death, which identifies curcumol as a promising therapeutic candidate for colon cancer [89].

Kaftan and collaborators investigated the potential of heteronemin (**32**), a marine‐derived sesterterpenoid natural product, to function as a ferroptotic agent in pancreatic cancer using the Panc‐1 cell line (Figure 13). Compound **32** showed potent cytotoxicity in in vitro assays, with IC_50_ values of 55 nM for Panc‐1 cells and 256 nM for HaCaT cells after 48 h of treatment, indicating a good selectivity index for pancreatic cancer cells. Mechanistic studies revealed that treatment with **32** significantly raised lipid peroxidation, a hallmark of ferroptosis, and modulated the expression of key proteins involved in autophagy and ferritin‐mediated iron storage. Compound **32** disrupts iron homeostasis and the redox balance to overcome cell survival adaptations in pancreatic cancer. Collectively, these results suggest that **32** is a promising therapeutic candidate from marine resources and suggest that targeting ferroptosis‐related pathways may be a strategy to improve the clinical management of pancreatic malignancies [90].

**FIGURE 13:**
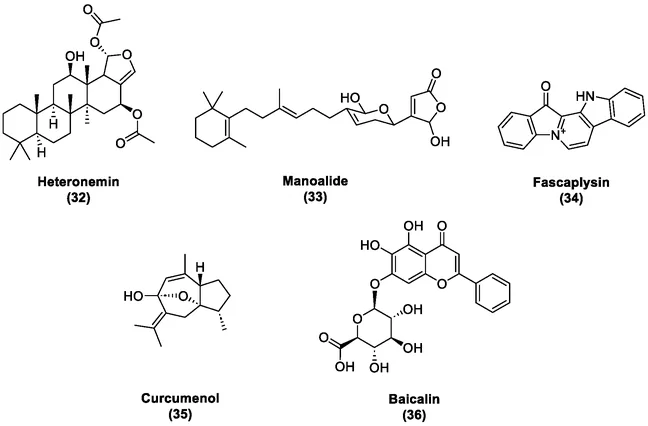
Natural products with anticancer activity, **32**–**36**.

Manoalide (**33**), a marine natural product and known phospholipase A2 (PLA2) inhibitor, was biologically evaluated by Ni and colleagues, who identified its potential as an anticancer agent in promoting epidermal growth factor receptor‐tyrosine kinase inhibitor (EGFR‐TKI) sensitivity in nonsmall‐cell lung cancer (NSCLC) (Figure 13). Cytotoxicity and viability assays showed that **33** significantly inhibited the proliferation of kirsten rat sarcoma viral oncogene homolog (KRAS)‐mutated (A549 and H157) and EGFR‐mutated (HCC827 and PC9) lung cancer cell lines and Kras^G12D^‐driven primary lung cancer organoids. In an acquired osimertinib‐resistant model (HCC827OR) with an increase in IC_50_ for osimertinib from 0.01 μM (parental) to 6.01 μM, co‐treatment with **33** restored drug sensitivity. Mechanistic studies demonstrated that **33** induces endoplasmic reticulum (ER) stress, as evidenced by cellular vacuolation and upregulation of GRP78, protein kinase R‐like endoplasmic reticulum kinase (PERK), and IRE1α, driven mainly by mitochondrial ROS accumulation. This increased oxidative stress promoted lipid peroxidation and inhibited mitochondrial fatty acid oxidation, resulting in the accumulation of intracellular lipid droplets. Furthermore, the ROS produced by **33** suppressed the KRAS‐ERK signaling pathway and thus sensitized KRAS‐mutated cells to the targeted therapy. In the meantime, **33** inhibited the NRF2‐SLC7A11 antioxidant axis and promoted mitochondrial Ca^2+^ overload to induce ferroptosis, which led to the downregulation of FTH1 and subsequent intracellular iron accumulation. Together, these results indicate that **33** is a multi‐modal EGFR‐TKI sensitizer that overcomes drug resistance in aggressive lung cancers by coordinated disruption of redox homeostasis, mitochondrial calcium balance, and lipid metabolism [91].

Luo and coworkers reported the biological evaluation of fascaplysin (**34**), a natural product from the marine sponge, and its anticancer mechanisms in NSCLC (Figure 13). Cytotoxicity assays were performed using human NSCLC cells (A549), with an IC_50_ value of 2.923 µM, and additional biological assays showed inhibition of cell migration related to the suppression of the Wnt/β‐catenin signaling pathway and the reversal of the epithelial‐mesenchymal transition (EMT) phenotype. Mechanistic studies showed that **34** induced G0/G1 cell cycle arrest and intrinsic mitochondrial apoptosis induction, as demonstrated by the loss of mitochondrial membrane potential, downregulation of BCL‐2, up‐regulation of BAX, and increased ROS production. Concurrently, **34** also triggered ferroptosis, as evidenced by the intracellular Fe^2+^ accumulation, increased lipid ROS, and mitochondrial structural abnormalities. This effect was associated with the downregulation of GPX4 and FTH1 and with the atypical upregulation of SLC7A11. Altered SLC7A11 expression correlated with the activation of the ER stress response, as demonstrated by the upregulation of ATF4, HSPA5, and CHOP, suggesting an ATF4‐dependent regulatory mechanism mediating ferroptotic vulnerability. Moreover, **34** markedly increased the expression of programmed cell death ligand 1 (PD‐L1) both in vitro and in an LLC syngeneic mouse model. In vivo experiments showed that this PD‐L1 upregulation sensitized tumors to anti‐PD‐1 immunotherapy, which significantly inhibited tumor growth and increased intratumoral infiltration of CD4^+^ and CD8^+^ T cells. In summary, these findings point to **34** as a multimodal cell death inducer that acts via a concerted mechanism involving ROS‐mediated apoptosis and ER stress‐associated ferroptosis. Our results identify **34** as a promising natural scaffold for the treatment of NSCLC and for improving the efficacy of immune checkpoint blockade therapies [92].

Zhang and collaborators reported the biological evaluation of curcumenol (**35**), an active sesquiterpenoid isolated from *Curcuma wenyujin*, as a potent inducer of ferroptosis in lung cancer (Figure 13). Cytotoxicity and colony‐formation assays showed that **35** dose‐dependently inhibited the proliferation and viability of human lung cancer cell lines (H1299 and H460) with low toxicity to normal lung epithelial cells. Mechanistic studies indicated that **35** mainly induced ferroptosis but not apoptosis, necroptosis, or autophagy, as cell death was efficiently rescued by specific ferroptosis inhibitors including DFO, ferrostatin‐1, and liproxstatin‐1. Ferroptotic cell death was characterized by the depletion of intracellular GSH, increased accumulation of ROS and MDA, and increased labile iron pool Fe^2+^. RNA sequencing‐based transcriptomic profiling further revealed that **35** significantly downregulates the expression of lncRNA H19. Molecular analyses revealed that lncRNA H19 functions as a ceRNA to sponge miR‐19b‐3p, and **35** downregulates H19 expression, resulting in the release of miR‐19b‐3p, which targets and suppresses the translation of FTH1. The suppression of FTH1 causes iron storage dysfunction, which aggravates oxidative stress and lipid peroxidation. These in vitro results were confirmed in vivo, using a subcutaneous H460 xenograft mouse model, where **35** administration (200 mg.kg^–1^.day^–1^) profoundly inhibited tumor growth, and this effect was reversed by co‐treatment with DFO. In conclusion, these findings indicate that **35** exerts its anticancer effect by co‐regulating the lncRNA H19/miR‐19b‐3p/FTH1 signaling axis to induce ferroptosis vulnerability and suggest that **35** may be a potential targeted therapeutic agent for lung cancer [93].

Kong and collaborators reported the biological evaluation of baicalin (**36**), which is a bioactive flavonoid extracted from *Scutellaria baicalensis* Georgi, demonstrating its potent antitumor efficacy against bladder cancer cells (Figure 13). Cytotoxicity assays showed that **36** inhibited the viability and proliferation of human bladder cancer cell lines (5637 and KU‐19‐19) in a dose‐dependent manner. Mechanistic studies showed that **36** primarily triggered ferroptosis, an iron‐dependent RCD characterized by intracellular accumulation of ROS and elevation of the labile iron pool. Through detailed molecular analysis, FTH1 was identified as a key upstream regulator of this process. The results showed that **36** treatment significantly downregulates the expression of FTH1, disrupting iron storage and promoting oxidative lipid peroxidation. Importantly, overexpression of FTH1 or pharmacological rescue with the iron chelator DFO efficiently rescued the cells from **36**‐mediated cytotoxicity, while inhibitors of apoptosis, autophagy, and necroptosis did not prevent cell death. Taken together, these findings reveal **36** as a potent natural inducer of FTH1‐dependent ferroptosis and suggest its therapeutic potential as a novel treatment approach for bladder cancer [94].

### 4.5 Modulators of NRF2, Oxidative Stress Response, and Auxiliary Antioxidant Enzymes

NRF2 is a master transcriptional regulator that orchestrates cellular defense against oxidative stress by activating antioxidant (ARE)‐dependent genes, including HO‐1/HMOX1, NQO1, peroxiredoxins (PRDXs), and enzymes involved in GSH and biopterin metabolism. Secondary antioxidant barriers are used by tumor cells to resist ferroptosis. The NRF2/ARE signaling cascade and its downstream target enzymes can be suppressed or modulated to abolish these barriers. This subsection describes natural agents against NRF2 signaling and the associated auxiliary antioxidant network components, disabling the oxidative stress buffering capacity and favoring lethal lipid peroxidation.

Tian and colleagues characterized new unexplored biosynthetic gene clusters in another study by the genomic analysis of eight *Kibdelosporangium* strains. Of *K. phytohabitans* XY‐R10, two angucyclines (**37** and **38**) and the other six azicemicins (**39**–‍**44**), eight novel natural products have been isolated from this organism and analyzed. Their structures were characterized by detailed spectroscopic analyses, single‐crystal X‐ray diffraction, and electronic circular dichroism. The ferroptosis protective effects of compounds **37**–**44** were investigated at 5 μM in A375 and H1299 cells with RSL3 as a ferroptosis inducer. Of these compounds, compound **39** was found to have strong protection against ferroptosis. It was observed that RSL3 induced lipid peroxidation to a large degree in A375 cells, followed by a significant attenuation of the toxicity by compound **7**. Structurally, RSL3 promoted the action of the heme oxygenase 1 gene (HMOX1), a stress‐response gene related to the ferroptosis response; however, the presence of compound **39** attenuated this induction. Inhibition of the pro‐oxidative cell‐oxidative state through reduction of the activity of the inhibitory protein GTP cyclohydrolase 1 (GCH1), which is a critical antioxidant capacity gene, was reversed by compound 8, as revealed by RSL3. From these results, it was inferred that compound **39** has anti‐ferroptosis activity by regulating oxidative stress‐related pathways such as HMOX1 repression and GCH1 expression restoration. Compounds **37**–**44** were also explored for their antiproliferative activities against five cancer cell lines (K562, Huh7, HeLa, A549, and HCT116). Only compound **42** showed significant cytotoxicity, with IC_50_ values lower than 10 μM in the majority of cell lines, except for K562 (Table 1) [95].

**TABLE 1:** Antiproliferative activity (IC_50_, μM) of angucyclines (37–38) and azicemicins (39–44) isolated from *Kibdelosporangium phytohabitans* XY‐R10 against selected human cancer cell lines.

Compound	Structure	IC_50_, μM			
		Huh7	HeLa	A549	HCT116
**37**	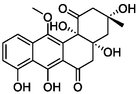	>30	>30	>30	>30
**38**	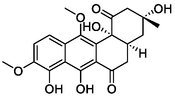	>30	>30	>30	>30
**39**	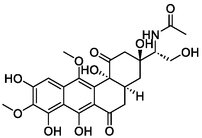	>30	>30	>30	>30
**40**	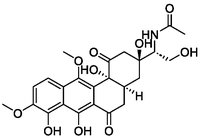	>30	>30	>30	>30
**41**	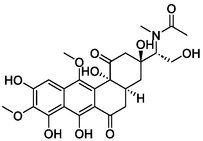	>30	>30	>30	>30
**42**	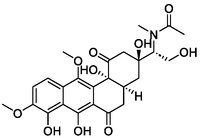	6.33	2.44	7.05	4.76
**43**	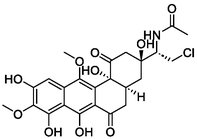	>30	>30	>30	>30
**44**	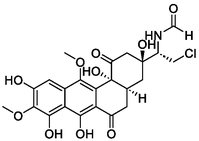	>30	>30	>30	>30
**Taxol**	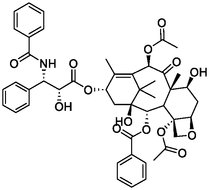	0.00443	0.00297	0.00173	0.00181

In another interesting study, wedelolid A (**45**) is being investigated as an antitumor agent among sesquiterpene lactones in gastric cancer (Figure 14). This compound **45** had cytotoxicity at IC_50_ values ranging from 2.81 to 8.80 μM in SGC‐7901, AGS, MGC‐803, BGC‐823, and KATO‐III from gastric cancer cell lines, while GES‐1 it was found to be less toxic (IC_50_ = 13.8 μM), suggesting a certain selectivity toward cancer. Specifically, gastric cancer cells were more sensitive than cisplatin to compound **45** (IC_50_ = 8.61–28.49 μM) but not to BGC‐823. With the exception of the BGC‐823 cell line, **45** caused both apoptosis and ferroptosis through mechanisms. The apoptotic cell death was observed by activation of caspases, cleavage of poly(ADP‐ribose) polymerase 1 (PARP1), and influence of the BCL‐2/BAX ratio. In parallel, compound **45** induced ferroptosis, accompanied by high ROS production, resulting in lipid peroxidation, as well as a depletion of cellular antioxidant defenses mediated by inhibition of the X_c_^−^/GPX4 axis. Moreover, **45** initiated the Keap1/NRF2/HO‐1 pathway, enhancing oxidative stress and inducing cell apoptosis. The simultaneous induction of apoptosis and ferroptosis also points to a synergistic mechanism that underlies its anticancer action. In vivo, **45** suppressed tumor growth in an MGC‐803 xenograft model with a tumor weight inhibition rate of 45.17% at 5 mg·kg^–1^, compared to 66.56% (2 mg·kg^–1^) for cisplatin. Significantly, compound **45** demonstrated relatively low systemic toxicities, thus indicating its potential application as a therapeutic agent in gastric cancer [96].

**FIGURE 14:**
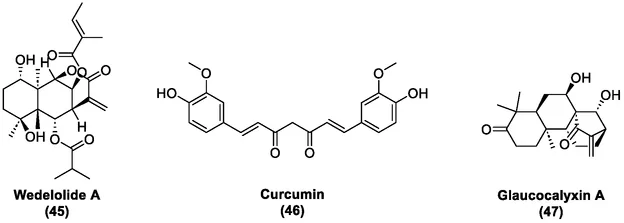
Natural products with anticancer activity, **45**–**47**.

Yuan and collaborators showed that curcumin modulates the PERK/NRF2/HO‐1 signaling pathway to cause ferroptosis in HCC (Figure 14). Curcumin, **46**, a naturally occurring substance that has been shown to have anticancer effects on a variety of tumor types, has drawn a lot of interest for additional research. After 48 h of treatment, curcumin significantly suppressed HCC cells (HepG2 and PLC), with IC_50_ values of 30.86 and 25.08 μM, respectively; ferrostatin‐1 and DFO reversed this effect. After curcumin treatment, metabolic analyses of HCC cells showed elevated levels of total iron, Fe^2+^, MDA, and ROS, as well as decreased levels of GSH. Ferrostatin‐1 attenuated these effects. Curcumin also dramatically reduced the expression of GPX4 while increasing the expression of HO‐1, NRF2, and PERK, according to Western blot analysis. Additionally, the **46**‐mediated inhibition of HCC cell viability was partially reversed, and the decrease in GPX4 expression was lessened by the use of PERK, HO‐1, and ER stress inhibitors. These results demonstrate curcumin’s potential as a therapeutic agent that targets ferroptotic pathways in HCC and show that it causes ferroptosis by activating the PERK/NRF2/HO‐1 axis. The mechanism by which curcumin causes ferroptosis in HCC cells was discovered in this study. The results lay the groundwork for further research on curcumin’s potential therapeutic use in HCC. Even though curcumin’s mechanisms of action have been greatly clarified, more in vitro and in vivo research is still needed for further validation [97].

Targeting peroxiredoxin 1 (PRDX1) and thioredoxin reductase 1 (TXNRD1) in NSCLC, the natural product glaucocalyxin A (**47**) was found to induce autophagy‐mediated ferroptosis (Figure 14). Compound **47** has a wide range of pharmacological activities and is one of the active ingredients in Weifuchun, a traditional Chinese medicine formulation that is frequently used in clinical settings to treat precancerous gastric lesions. Nevertheless, little is known about the specific molecular mechanisms that underlie its action against NSCLC and its cellular targets. Compound **47** showed IC_50_ values ranging from 2.50 to 8.38 μM in cell lines (MGC803, LLC, A549, and Calu‐1), with A549 cells showing the strongest antiproliferative activity. Interestingly, compared to gastric cancer cells, its in vitro activity against NSCLC cell lines was higher. Compound **47** treatment at a dose of 20 mg·kg^–1^ significantly inhibited the growth of subcutaneous tumors in C57BL/6 and nude mouse models, as demonstrated by decreased tumor size and decreased expression of proliferating cell nuclear antigen. Additionally, hematoxylin and eosin (H&E) staining and stable body weight in naked mice showed no discernible organ toxicity. Compound **47** is covalently bound to Cys173 of PRDX1 and to Cys59, Cys497, and Cys498 of TXNRD1 to inhibit antioxidant activity. Proton NMR spectroscopy, which showed that L‐cysteine mainly goes through a Michael addition reaction with the α,β‐unsaturated ketone moiety in compound **47’s** structure, corroborated these conclusions. Significant intracellular ROS accumulation resulted from this dual‐target inhibition’s notable disruption of cellular redox homeostasis. The ensuing oxidative stress caused autophagic pathways to be activated, which in turn caused NSCLC cells to undergo ferroptosis. All things considered, this research validates compound **47’s** strong inhibitory activity against NSCLC in vitro and in vivo and offers a new molecular mechanism for its therapeutic effects [98].

Wang and coworkers conducted a pharmacological study on a natural naphthoquinone, juglone (**48**), to assess its anticancer potential in HCC (Figure 15). The study found significant cytotoxicity of the compounds in PLC and Huh7 cell lines, with IC_50_ values of 16 and 11 μM, respectively. Further phenotypic evaluation showed that treatment with 10 μM **48** effectively reduced cell proliferation, migration, and invasion and induced cell cycle arrest at the G0/G1 phase. Mechanistic evaluations indicated that **48** induced ferroptosis through the FOSL1–HMOX1 signaling axis, where FOSL1 acted as a transcriptional activator of HMOX1, which subsequently induced ferroptotic cell death. In this process, increased intracellular iron content, increased lipid peroxidation, and depletion of GSH are involved. In vivo experiments confirmed that intratumoral administration of **48** (2 mg·kg^–1^ day^–1^) significantly suppresses tumor progression. Our findings identify the FOSL1–HMOX1 axis as a critical regulatory node of ferroptosis in HCC and suggest **48** as a promising therapeutic candidate for HCC and pan‐cancer clinical applications [99].

**FIGURE 15:**
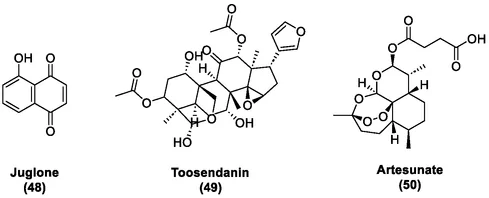
Natural products with anticancer activity, **48**–**50**.

Yang and colleagues reported the investigation of toosendanin (**49**), a natural product isolated from *Melia toosendan*, and its antitumor efficacy in HCC (Figure 15). Researchers demonstrated that **49** exerts potent antiproliferative effects via modulation of the WW domain‐containing oxidoreductase (WWOX) by inducing ferroptosis in HCC cells. Mechanistic studies indicate that **49** acts as a multimodal regulator of ferroptosis by targeting two distinct signaling axes, the WWOX‐p53/SAT1 axis and the WWOX‐NRF2/FPN1 axis. **49**‐induced activation of WWOX enhances the transcriptional activity of p53, which leads to the upregulation of SAT1, promoting ferroptosis and iron‐dependent oxidative stress. Meanwhile, **49** promotes the ubiquitination and degradation of NRF2 and inhibits the expression of FPN1 (SLC40A1) and GPX4, leading to the accumulation of intracellular Fe^2+^ and lipid peroxides. These results were further supported in vivo in xenograft models, where **49** effectively inhibited tumor growth through the activation of these ferroptotic pathways. Altogether, these findings indicate that **49** acts as a potential therapeutic candidate for HCC by disrupting redox homeostasis and iron metabolism through WWOX to induce ferroptotic cell death. Here, they show that modulation of the WWOX‐NRF2/p53 crosstalk may represent a potential approach to overcome the therapeutic limitations in HCC [100].

Artesunate (**50**), a natural sesquiterpene lactone derived from *Artemisia annua*, was investigated for its anti‐melanoma efficacy by Liu and coworkers (Figure 15). The study showed that **50** markedly inhibits the proliferation of melanoma cells and triggers ferroptotic cell death. Mechanistic studies demonstrated that **50** directly targets Ido1 and thus represses hypermethylated in cancer 1 (Hic1)‐mediated transcriptional repression of HMOX1, leading to ferroptosis of melanoma cells. Importantly, **50** is selective, as it does not induce ferroptosis in CD8^+^ T cells due to the low HMOX1 expression. In addition, **50**‐mediated inhibition of Ido1 leads to increased tryptophan levels, which inhibit nuclear factor of activated T cells 1 (NFATc1)‐mediated programmed cell death protein 1 (PD‐1) transcription, thus promoting CD8^+^ T cell activation and antitumor immunity. The in vivo and in vitro experiments showed that **50** may simultaneously enhance the synergistic therapeutic effect by inducing ferroptosis of tumor cells and enhancing the immune response of the host. These collectively identify **50** as a promising therapeutic for melanoma, providing a novel mechanistic rationale for its function as an Ido1 inhibitor and inducer of ferroptosis in cancer immunotherapy [101].

Ma and coworkers have reported the systematic investigation of daphnetin (**51**), a natural coumarin derivative isolated from *Daphne koreana* Nakai, for its antitumor efficacy in ovarian cancer (Figure 16). In vitro assays showed that **51** significantly suppresses proliferation, migration, and invasion of ovarian cancer cell lines. Mechanistic studies showed that **51** induced ferroptosis, an iron‐dependent form of RCD, as evidenced by iron accumulation and increased lipid peroxidation. Molecular studies identified NAD(P)H quinone dehydrogenase 1 (NQO1) as a direct molecular target of daphnetin. **51** inhibits NQO1, which disrupts the cellular antioxidant defense system, leading to the accumulation of ROS and ferroptotic cell death. Furthermore, **51** increased the sensitivity of ovarian cancer cells to cisplatin. In aggregate, these findings show that **51** is a potent pro‐ferroptotic agent in ovarian cancer and suggest that targeting the NQO1‐mediated redox axis could serve as a promising therapeutic strategy to overcome chemoresistance and improve outcomes in ovarian cancer management [102].

**FIGURE 16:**
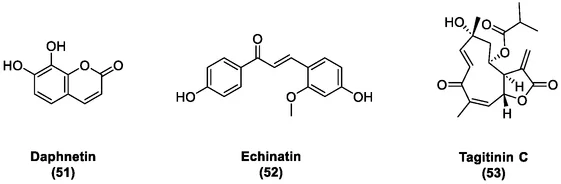
Natural products with anticancer activity, **51**–**53**.

Kang and coworkers conducted a systematic investigation of echinatin (**52**), a natural chalcone isolated from *Glycyrrhiza* species, for its antitumor efficacy in cutaneous squamous cell carcinoma (Figure 16). **52** induced ferroptotic cell death and significantly inhibited tumor growth in both in vitro and in vivo models. Mechanistic studies revealed that **52** directly targets glutathione S‐transferase mu 3 (GSTM3), interfering with its protective role against oxidative stress. Specifically, **52**‐mediated inhibition of GSTM3 decreases the antioxidant enzyme peroxiredoxin 2 (PRDX2), resulting in the accumulation of ROS and lipid peroxidation. These findings were further validated in UV‐‍induced cutaneous squamous cell carcinoma mouse models and patient‐derived xenografts, confirming that **52** induces ferroptosis via the GSTM3/PRDX2 axis. Collectively, this work establishes **52** as a promising therapeutic candidate for cutaneous squamous cell carcinoma management and provides a new mechanistic paradigm for targeting ferroptotic vulnerabilities via modulation of GSTM3‐mediated redox homeostasis [103].

The biological evaluation of the natural sesquiterpene lactone tagitinin C (**53**) for its potent antitumor efficacy in colorectal cancer cells was reported by Wei and coworkers (Figure 16). Results of cytotoxicity assays indicated the potent antiproliferative effect of **53** on colorectal cancer cell lines. Mechanistic studies showed that the compound induces ferroptosis, an iron‐dependent form of programmed cell death characterized by the accumulation of lipid ROS. The molecular analyses revealed that **53** activated the PERK‐NFR2‐HO‐1 signaling axis. This pathway, which is usually a stress‐response mechanism, is activated and leads to the upregulation of HO‐1, which ultimately exacerbates oxidative stress and facilitates ferroptotic cell death in the context of colorectal cancer. In combination, these findings identify **53** as a novel ferroptosis inducer in colorectal cancer and highlight the significance of the PERK‐NRF2‐HO‐1 pathway in mediating **53** cytotoxicity, which may provide a therapeutic target for colorectal cancer treatment [104].

### 4.6 Specific Organellar Regulators, p53‐GSH Axis, and Upstream Ferroptosis Modulators

Further to the canonical System X_c_^–^/GPX4 axis, susceptibility to ferroptosis parallels cell‐survival metabolic rewiring and organellar signaling pathways. Independent suppressor networks, such as the FSP1‐CoQ10 pathway, mitochondrial membrane channels (voltage‐dependent anion channel 2, VDAC2), chaperone‐mediated protein stability (heat shock protein 70, HSP70), and p53‐dependent GSH depletion, heavily influence the execution of ferroptotic death. This subsection addresses natural compounds targeting these alternative suppressor pathways, organellar integrity, or regulatory protein networks to induce lipid ROS accumulation and overcome therapeutic resistance in aggressive tumors.

Liu and coworkers reported that nidurufin (**54**) exhibited antiproliferative activity against prostate cancer (Figure 17). The lead compound also displayed significant cytotoxic activity against androgen receptor (AR)‐positive prostate cancer cells, particularly the 22Rv1 cell line, with an IC_50_ value of 10.30 μM. In mechanistic studies, it was shown that **54** induces ferroptosis, as evidenced by mitochondrial shrinkage and increased intracellular ROS levels. Notably, **54** did not directly bind to AR but rather interacted with HSP70, which interfered with its chaperone function. This interaction destabilized the AR protein and inhibited AR‐mediated transcriptional activity. Thus, the expression of membrane‐bound *O*‐acyltransferase domain‐containing protein 2 (MBOAT2), a downstream AR target implicated in the suppression of ferroptosis, was dramatically downregulated. In vivo studies, conducted on a zebrafish xenograft model, also proved that **54** significantly inhibited tumor growth and metastasis. Taken together, these findings demonstrate an unexplored signaling cascade that mediates the interplay between protein homeostasis, AR signaling, and ferroptotic cell death [105].

**FIGURE 17:**
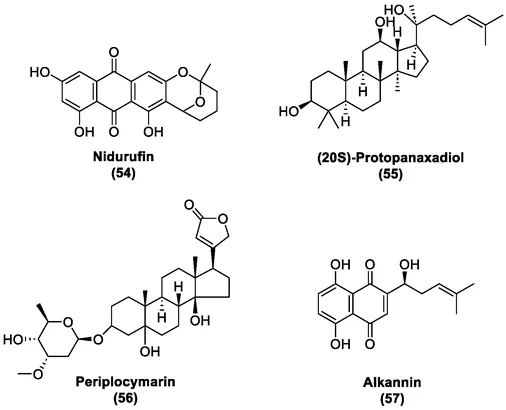
Natural products with anticancer activity, **54**–**57**.

A study by Liu and coworkers examined a ginsenoside derivative, (20*S*)‐protopanaxatriol (**55**), as a ferroptosis inducer in HCC and cholangiocarcinoma by targeting FSP1 (Figure 17). They initially screened a library of 180 natural product‐derived compounds, finding compound **55** to be a strong ferroptosis inducer with a good safety profile both in vitro and in vivo. Compound **55** induces ferroptosis by targeting the plasma membrane‐localized CoQ10 oxidoreductase FSP1. This inhibition increases ACSL4‐dependent arachidonic acid oxidation and boosts mitochondrial ROS production, thus improving ferroptosis. Importantly, ferroptosis induced by **55** happens independently of the SLC7A11–GSH–GPX4 pathway. Further evidence showed that just inhibiting FSP1 was enough to trigger ferroptosis in some HCC and cholangiocarcinoma cells. Additionally, combining the inhibition of FSP1 and γ‐glutamylcysteine synthetase (GCS) worked together to induce ferroptosis in resistant cancer cells without affecting noncancerous cells. These results indicate the need to reevaluate the role of FSP1 in ferroptosis and emphasize its potential as a main therapeutic target in certain cancer subtypes. The findings reveal a previously unrecognized function of FSP1 in inducing ferroptosis and highlight the potential benefits of targeting FSP1 and GCS together in these cancers. The in vivo antitumor effects of combining **55** and buthionine sulfoximine (BSO) were tested in a Hep3B xenograft tumor model. Neither **55** nor BSO alone effectively stopped tumor growth. However, their combination significantly reduced tumor progression without causing any weight loss in the subjects. Importantly, this effect vanished when ferrostatin‐1 was given at the same time, confirming the induction of ferroptosis in vivo. However, some limitations exist. The exact mechanisms through which FSP1 inhibition leads to mitochondrial dysfunction are not fully understood. Also, while BSO was found to work synergistically with FSP1 inhibitors, more research is needed to identify other factors that may enhance ferroptosis induced by FSP1 inhibitors. Overall, these findings stress the importance of FSP1 as a key target for inducing ferroptosis and provide strong support for developing FSP1‐based cancer treatments [106].

Ke and colleagues conducted a pharmacological study on periplocymarin (**56**), a cardiac glycoside derived from the traditional plant *Periploca sepium*, to assess its ability to induce ferroptosis in gastric cancer (Figure 17). Cytotoxicity testing on human gastric cancer cell lines (specifically HGC27 and MKN45) demonstrated that **56** causes a gradual apoptotic response by inhibiting glycolysis, but when combined with ferroptosis inducers (like RSL3 and erastin) or cisplatin, it leads to rapid and intense ferroptotic cell death both in vitro and in vivo. According to mechanistic investigations, **56** binds directly to the α1 subunit of the Na^+^/K^+^‐ATPase (ATP1A1), which in turn triggers the activation of proto‐oncogene tyrosine‐protein (Src) kinase. This activation leads to the direct tyrosine phosphorylation of the yes‐associated protein/transcriptional co‐activator with PDZ‐binding motif (YAP/TAZ) without involving the Hippo pathway, which facilitates their movement to the nucleus and their subsequent interaction with the TEAD transcription factor. The YAP/TAZ‐TEAD complex subsequently interacts directly with the promoter region of the TRF1 gene (located 401–409 nucleotides upstream of the transcription start site), leading to an increase in TFRC transcription. This upregulation, in turn, boosts the influx of intracellular Fe^2+^, raises the levels of lipid peroxidation, and heightens susceptibility to ferroptosis. These findings, when taken together, indicate that the regulatory axis of ATP1A1‐Src‐YAP/TAZ‐TFRC plays a vital role in determining sensitivity to ferroptosis, emphasizing the promise of using cardiac glycosides such as **56** as powerful ferroptosis sensitizers in combination cancer therapies [107].

Yu and colleagues assessed the antitumor efficacy of alkannin (**57**), a quinonoid compound isolated from *Lithospermum erythrorhizon*, in gastric cancer (Figure 17). In vitro assays demonstrated that AGS and HGC27 cell lines exhibited potent cytotoxicity with calculated IC_50_ values of 0.7643 and 0.9989 μM, respectively, and significant inhibition of cell proliferation, migration, and invasion. Mechanistic studies showed that **57** suppressed the c‐Fos/SREBF1 signaling axis and the expression of major genes involved in lipid metabolism. This metabolic disturbance results in perturbation of cell membrane integrity and redox homeostasis, leading to apoptosis, as exemplified by modulation of BCL‐2, Bax, and caspase‐3 cleavage, and ferroptosis, which involves buildup of ROS and lipid peroxidation. In vivo in xenograft models, **57** administration significantly reduced tumor volume and weight without significant systemic toxicity. In summary, our study confirms the c‐Fos/SREBF1 axis as a promising therapeutic target and **57** as a potent agent for the management of aggressive gastric malignancies by harnessing the lipidomic vulnerabilities of cancer cells [108].

Chen and colleagues evaluated the antitumor activity of isoliensinine (**58**), a natural bisbenzylisoquinoline alkaloid, in lung adenocarcinoma (Figure 18). **58** showed significant cytotoxic activity in lung adenocarcinoma cells in vitro, with IC_50_ values of 7.21 μM (A549 cells) and 6.18 μM (H1299 cells) after 24 h of treatment, demonstrating a potent suppression of cell proliferation. Mechanistic studies identified apurinic/apyrimidinic endodeoxyribonuclease 1 (APEX1) as a direct molecular target of **58**. Computational docking and CETSA confirmed the stable interaction of the compound with APEX1. Binding of **58** causes degradation of APEX1, resulting in a significant increase in intracellular ROS production, oxidative stress, and apoptosis. Furthermore, in vivo xenograft mouse models demonstrated that **58** treatment significantly inhibited tumor growth and volume without systemic toxicity. Collectively, these results suggest that **58** is a potent pharmacological inhibitor of APEX1‐driven survival pathways in lung cancer. The present study suggests **58** as a promising natural scaffold for the rational design of targeted therapies in lung adenocarcinoma based on its capacity to perturb redox homeostasis at sub‐micromolar concentrations.

**FIGURE 18:**
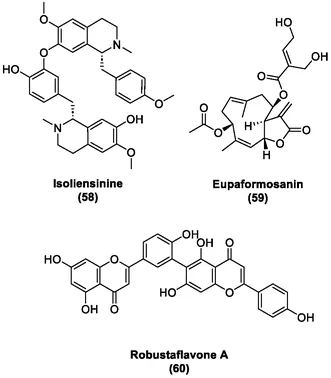
Natural products with anticancer activity, **58**–**60**.

Wei and coworkers reported the biological evaluation of eupaformosanin (**59**), a natural sesquiterpene lactone isolated from *Eupatorium cannabinum* Linn., and identified its potent antitumor efficacy in TNBC (Figure 18). Cytotoxicity assays in several TNBC cell lines showed that **59** significantly inhibits cell viability in a dose‐dependent fashion. Mechanistic studies showed that **59** triggers a multimodal cell death program combining mitochondrial apoptosis, indicated by collapsing mitochondrial membrane potential (ΔΨ_m_) and increased mitochondrial ROS generation, and ferroptosis, as determined by GSH depletion, intracellular Fe^2+^ accumulation, and significant lipid peroxidation. Molecular analysis suggests that **59** mediates site‐specific ubiquitination and subsequent proteasomal degradation of mutant p53. Mutant p53, which frequently functions as an oncogene with gain‐of‐function properties that promote cell survival and resistance to stress in TNBC, is effectively depleted by **59**, sensitizing malignant cells to oxidative stress and promoting programmed cell death. Together, these findings identify **59** as a novel regulator of mutant p53 protein and suggest that targeted degradation of oncogenic p53 variants represents a promising therapeutic approach to counteract the intrinsic aggressiveness of TNBC [109].

Xie and colleagues isolated and biologically evaluated robustaflavone A (**60**), a novel naturally occurring biflavonoid isolated from *Selaginella trichoclada*, which showed its potent antitumor efficacy against human breast cancer (Figure 18). The cytotoxicity assays showed that **60** has a strong antiproliferative activity on the MCF‐7 breast cancer cell line, efficiently inhibiting cell viability with an IC_50_value of 11.89 μM. Mechanistic studies demonstrated that **60** induced nonapoptotic cell death mainly through ferroptosis, as evidenced by a significant increase in intracellular ROS, a decrease in mitochondrial cristae, and widespread lipid peroxidation. Extensive molecular and transcriptomic analyses suggested that **60** induces ferroptosis through a mitochondria‐dependent pathway. Molecular docking and protein expression profiling showed that **60** interacts with E3 ubiquitin ligase Nedd4 (neural precursor cell expressed developmentally downregulated 4) and suppresses its expression, resulting in inhibition of ubiquitination and proteasomal degradation of VDAC2. The up‐regulation and accumulation of VDAC2 then promote mitochondrial dysfunction, oxidative stress, and ultimately ferroptotic cell death. Together, these results establish **60** as a promising candidate for natural breast cancer therapy and suggest that targeted modulation of the Nedd4/VDAC2 axis may be a novel approach to induce ferroptosis in malignant cells [110].

## 5 Conclusions and Future Perspectives

The induction of ferroptosis has been proposed as a promising therapeutic strategy for aggressive, metastatic, and otherwise resistant cancers. Tumor cells such as TNBC and gliomas are sensitive to lethal lipid peroxidation [32, 111] because of the increased intracellular iron levels and high reliance on antioxidant systems to overcome oxidative stress. Therapeutic failure due to the evasion of apoptosis calls for the search for alternative routes of controlled cell death. In this context, the present review summarizes the main findings related to the involvement of ferroptosis and the therapeutic applications of natural bioactive compounds.

At this frontier, natural products are significant in that they provide a rich source of lead structures for historically challenging pharmacological targets, such as GPX4 and NRF2 [44, 112, 113, 114]. Biogenic compounds, including artemisinin and derivatives, have been found to be efficient in disrupting iron homeostasis and inhibiting GPX4, often in synergy with apoptosis and autophagy [115]. Other phytochemicals, like curcuminoids, show bivalent mechanisms by inducing the expression of HO‐1 and reducing the levels of GPX4, resulting in selective lethality against malignant cells [32, 97, 116].

In this scenario, ferroptosis is an important mechanism for overcoming drug resistance. This form of nonapoptotic programmed cell death, associated with intracellular free iron overload and lethal peroxidation of membrane lipids, has emerged as an Achilles’ heel for aggressive tumors. Tumor cell lines of the mesenchymal transition, cancer stem cells (CSCs), and variants with multidrug resistance to conventional chemotherapeutics show an aggravated metabolic vulnerability to ferroptosis, making this pathway a highly relevant therapeutic target [46, 117].

Furthermore, the importance of natural products in preclinical research in oncology should be highlighted. Accumulated data show that natural products, especially polyphenols, flavonoids, terpenoids, and alkaloids, fill critical gaps that single‐target synthetic drugs often fail to do. These molecules exert multi‐target and multi‐pathway effects due to their evolutionary chemical diversity. They regulate iron metabolism (through the Fenton reaction), weaken the antioxidant defense system (by inhibiting the cystine/glutamate antiporter system X_c_^−^ or directly depleting the GPX4 enzyme), and induce strong oxidative stress in tumors [112, 118].

Secondary metabolites from plants and marine organisms have better preclinical safety profiles than first‐generation synthetic ferroptosis inducers (e.g., erastin), which often have limiting toxicity in vivo and have the ability to selectively kill hypermetabolic malignant cells over normal tissues [112].

The optimism justified by the strong findings in vitro and in animal models (in vivo) notwithstanding, the translation from the preclinical research laboratory to the patient bedside (bench to bedside) requires focusing scientific efforts on the following focal areas:

1. Nanotechnology and pharmacokinetic optimization: a major obstacle in the utilization of isolated natural products is their poor aqueous solubility, metabolic instability, and limited bioavailability. The future outlook is naturally linked to the advancement of smart drug delivery systems. The encapsulation of natural compounds within lipid nanoparticles, polymeric micelles, or targeted magnetic carriers is expected to increase the accumulation of such molecules in the tumor microenvironment, thus increasing local ferroptosis induction and diminishing peripheral side effects [117].
2. Combination therapies and synergism: the future of aggressive tumor management will not rely on monotherapy. An extremely logical strategy appears to be using natural products that modulate ferroptosis as sensitizing agents. These phytochemicals, combined with conventional chemo‐ or radiotherapy or immune checkpoint inhibitors (immunotherapy), can break down the antioxidant defenses of the tumor, reverse acquired resistance, and result in an immunogenically active tumor microenvironment by releasing ferroptosis‐induced damage‐associated molecular patterns (DAMPs) [117, 119].
3. Biomarker discovery: the identification and validation of in vivo response biomarkers are essential to propel ferroptosis‐based therapies into rigorous clinical trials. It will be important to map genetic signatures (e.g., expression of SLC7A11, GPX4, and FSP1) and monitor circulating lipid oxidation byproducts to select subgroups of patients with aggressive cancers who will receive personalized benefits from these nature‐based interventions [120, 121].

In conclusion, the interaction between ferroptosis biology and pharmacognosy represents a very promising frontier. Medical science is entering a new era in oncology, and the mechanisms by which the chemical richness of natural products orchestrates tumor lipid collapse are now being decoded. Not only does such a translational approach provide a feasible alternative to first‐generation chemotherapeutics but it also offers palpable and clinically manageable hope for the treatment of recalcitrant tumors that have hitherto been considered untreatable.

## Author Contributions

The author confirms sole responsibility for the following: conceptualization, methodology, investigation, data curation, formal analysis, visualization, writing – original draft, and writing – review and editing.

## Funding

This work was supported by the Coordenação de Aperfeiçoamento de Pessoal de Nível Superior (CAPES, financial code 001).

## Consent

The author has nothing to report.

## Conflicts of Interest

The author declares no conflicts of interest.

## Data Availability

The data that support the findings of this study are available from the corresponding author upon reasonable request.
